# On the photoluminescence in triarylmethyl-centered mono-, di-, and multiradicals

**DOI:** 10.3762/bjoc.21.80

**Published:** 2025-05-21

**Authors:** Daniel Straub, Markus Gross, Mona E Arnold, Julia Zolg, Alexander J C Kuehne

**Affiliations:** 1 OC III - Institute of Organic and Macromolecular Chemistry, Ulm University, Albert-Einstein-Allee 11, 89081 Ulm, Germany; 2 Center for Integrated Quantum Science and Technology, Ulm University, Albert-Einstein-Allee 11, 89081 Ulm, Germany

**Keywords:** Chichibabin hydrocarbon, COF, Gomberg radical, MOF, Müller hydrocarbon, Thiele hydrocarbon

## Abstract

Organic radicals with light-emitting properties and exceptional stability offer exciting opportunities to address spin-statistical limitations in organic electronics and advance quantum technologies. These radicals, acting as small molecular magnets, exhibit sensitivity to minute magnetic fields and can be tailored with diverse spin centers, making them ideal for spin-optical interfaces, representing key components in quantum communication systems. Furthermore, their ability to form organized, higher-dimensional assemblies presents a promising avenue for overcoming scalability challenges in quantum technologies. Despite their potential, achieving high luminescence quantum yields has largely been limited to donor-functionalized monoradicals, and a detailed understanding of the luminescent behavior of open-shell organic molecules remains elusive. This review delves into the photoluminescent properties and spin ground states of trityl-based mono-, di-, and multiradicals, examining the strategies employed to enhance their performance. Additionally, we review predictive methods for determining the luminescence and spin states of radicals, highlighting critical unresolved questions that must be addressed to unlock the full potential of trityl-based radicals in advanced technological applications.

## Introduction

**Figure 1:**
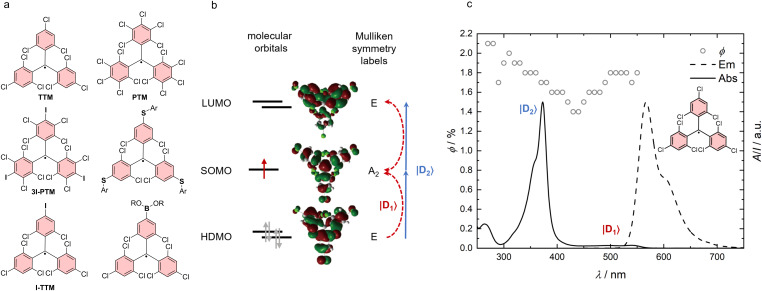
a) Tris(trichlorophenyl)methyl (**TTM**) radical and related trityl radicals, b) HDMO, SOMO, LUMO orbitals of the **TTM** radical with assigned Mulliken symmetry labels. c) Absorption, photoluminescence spectra and photoluminescence quantum yield ϕ of **TTM**. Figure 1b was adapted from [34], S. Sang et al., “Theoretical investigation of aromaticity and charge transfer in emission process of triarylmethyl radicals as OLED materials”, Quantum Chemistry, with permission from John Wiley and Sons. © 2020 Wiley Periodicals LLC. This content is not subject to CC BY 4.0.

Gomberg-type, triarylmethyl-centered radicals represent a class of stable organic radicals that exhibit luminescence. Typically, organic radicals that are luminescent exhibit poor photostability, and many representatives of the triarylmethyl family degrade upon photoexcitation [1–4]. For example, the photoluminescence of solutions containing perchlorotriphenylmethyl (**PTM**) or tris(2,4,6-trichlorophenyl)methyl (**TTM**) radicals degrades to half of their intensity within just a few minutes, while for their corresponding di- and multiradicals no fluorescence data is reported at all (see Figure 1a for chemical structures) [5–6].

However, research on fluorescent radicals has seen new impetus during the last decade, stimulated by the development of highly efficient electroluminescent devices based on donor-functionalized **TTM** radicals [7–13]. Today, new derivatives of donor-functionalized triarylmethyl radicals are being synthesized with enhanced photoluminescence quantum yield (ϕ) combined with improved photostability.

The spin-allowed transition from the excited doublet state to the ground state [7–13], combined with the absence of competing triplet states – which is a major pathway of efficiency loss in closed-shell emitters – promises to overcome the spin-statistical problem that limits the performance of conventional organic electronics. Without the chance to populate any dark triplet or quartet states, light-emitting radicals possess a theoretical internal quantum efficiency of up to 100% in electroluminescent devices [5,7–8,14].

While first examples of radical emitters in light-emitting diodes (LEDs) only delivered around 12% internal quantum efficiency [7], representing no improvement compared to conventional emitters, optimization of the donor-functionalized **TTM** radical and of the device geometry quickly allowed demonstration of LEDs with 100% internal quantum efficiency [8,12]. Modern approaches also incorporate light-emitting **TTM** radical derivatives into LEDs based on thermally activated delayed fluorescence to moderate excited triplet, doublet, and singlet states in an attempt to improve the overall device and emission performance [15].

Other prospective applications utilize the manipulation of the spin-state of the unpaired electron and the potential for optical readout of the spin state for quantum technological information processing, communication, or chemical and biological sensing [16–18].

However, there is a lack of understanding of why the absorption and photoluminescence of *D*_3_ symmetrical triphenylmethyl radicals are so weak in the visible spectrum. While it is understood that the functionalization with donor moieties breaks the *D*_3_ symmetry, the ϕ varies greatly among different donors and also when two or three donors are attached to the triarylmethyl radical center. Current approaches to improve the luminescence performance are limited to trial-and-error approaches or systematic screening of donors, while the underlying fundamental reasons for high or poor performance remain unclear.

The strong emission in some of the donor-functionalized triarylmethyl radicals has been explained by improved orbital overlap between the ground D_0_ and excited D_1_ states, while often geometrical alignment with a certain angle between the donor and the triarylmethyl units is being discussed. (The reader may note: electronic states are denoted in upright letters, e.g., D_0_, while symmetry descriptors are denoted in italics, e.g., *D*_3_*.*) Moreover, symmetry arguments are being employed to explain why certain transitions may be forbidden. This concept holds true for **TTM** and **PTM** with their *D*_3_ symmetries in comparison to triarylmethyl units that have been symmetry broken by the single substitution with a donor. However, simple symmetry arguments cannot explain why threefold-substituted triarylmethyl radicals have often comparable or higher ϕ than their monofunctionalized homologues. Also, the concept of intensity borrowing from higher excited states, due to orbital overlap, does not explain the improved performance sufficiently, especially when comparing the oscillator strengths that can be determined using density functional theory (DFT) studies [19–21]. Moreover, when looking at di- or multiradicals, there are no reports on substantial photoluminescence, nor are there concepts to increase the ϕ in such systems, despite the many examples of highly emissive donor-substituted **PTM**- and **TTM**-based mono-radicals.

In this overview, we will focus on reviewing the different hypotheses for the mechanisms governing the luminescence performance and we will provide examples and connections between reports, where the hypotheses have been tested and proven. We do not attempt to give a comprehensive overview of the entire research field of organic triarylmethyl-centered radicals, since there already exists a body of reviews covering the different molecular structures of mono-, di-, and multiradicals, synthetic approaches [22–25], state dynamics in luminescent radicals [26], and their application in light-emitting devices [27–29].

Instead, we aim to provide insight and understanding into the mechanisms and reasons behind the fluorescence color and the fluorescence quantum yield and derive structure–optical property relationships. We will provide the reader with an overview of the perspectives and challenges of open-shell light-emitting radicals and discuss what to do to solve the remaining problems in the future and prepare organic radicals for the challenges in emerging quantum technological applications.

## Review

### Triarylmethyl monoradicals

#### The importance of symmetry in triarylmethyl radicals

Gomberg’s radical is unstable at room temperature and dimerizes quickly to a closed-shell molecule. Only at temperatures as low as 90 K, the monoradical is stable, allowing recording of the absorption and emission spectra [30]. Symmetrical halogenation of the triphenylmethyl (or trityl) unit increases stability of the molecule, compared to the parent unsubstituted trityl radical first reported by Gomberg (see **TTM** and **PTM** in Figure 1a) [31]. Gomberg’s radical emits light in the green region of the visible spectrum, which is shifted bathochromically by substituting the *ortho*- and *para*-positions of the trityl phenyl rings with halogens. However, all symmetrical halo-trityl radicals exhibit low ϕ, usually below 3%. Such perhalo- or nonahalo trityl radicals exhibit a *D*_3_ propeller symmetry, which allows assigning of individual orbital geometries (see Figure 1b). Furthermore, such radicals follow the *alternant hydrocarbon* rule of the Hückel theory [32–33], which dictates that the highest singly occupied molecular orbital (SOMO) is non-bonding and energetically located exactly between the highest doubly occupied molecular orbital (HDMO) and the lowest unoccupied molecular orbital (LUMO) (see Figure 1b) [19].

In other words, in such *D*_3_ symmetrical alternant hydrocarbons the HDMO–SOMO and the SOMO–LUMO energy gaps are identical and their respective transitions exhibit identical transition dipole moments [19,32–33]. For the |D_1_⟩ transition these two degenerate transitions mix in an out-of-phase fashion leading to the observed weak absorption at 544 nm (see Figure 1b and c). When looking at the Mulliken symmetry labels, one realizes that the |D_1_⟩ transition is in fact symmetry forbidden. By contrast, in-phase mixing of the transitions for the |D_2_⟩ transition leads to strong absorption at 374 nm, a transition that is symmetry allowed (see Figure 1b and c).

In **TTM**, the ϕ is independent of the excitation wavelength (see Figure 1c). Excitation at 374 nm or 544 nm both lead to fluorescence with an identical fluorescence decay profile and identical ϕ of ≈2% (see Figure 1c). This behavior hints at the fact that the relaxed excited state, from which the emission occurs, is the same for excitation at 374 nm and 544 nm. Therefore, the higher energy for excitation to the D_2_ state must quickly be lost during relaxation to the relaxed D_1_ state, rendering the emission indistinguishable from the higher energy excitation.

While for **TTM** and **PTM** there is no transient absorption data to further elucidate the excited state dynamics, there is evidence for this fast relaxation in donor-functionalized **TTM** radicals [26].

Moreover, transient absorption has been employed to elucidate the formation of a non-luminescent side-product in trityl radicals. During photodegradation, two halogens are lost and two phenyl rings planarize to fuse to a fluorenyl unit, rendering the radical non-emissive [35–36].

According to “valence shell electron pair repulsion” (VSEPR) theory, one would expect a geometrical change of the **TTM** molecules from *D*_3_ to a pyramidal *C*_3_ symmetry upon excitation. However, for steric reasons the molecule remains mostly planar in the excited state [37]. As a result, we observe weak emission in the yellow to red spectrum, because the respective transition remains symmetry forbidden.

#### Circularly polarized photoluminescence

**PTM** has been developed prior to **TTM**, as the first stable triphenylmethyl radical [38–39]. In contrast to the Gomberg radical, the perchlorination prevents dimerization through the *para*-position. Moreover, the chlorine substituents in the *ortho*-positions twist the phenyl rings into a propeller conformation and out of the sp^2^-hybridization plane of the central methine radical unit. Now, the chlorine substituents screen the hemispheres above and below this plane, protecting the unpaired electron in the p-orbital from oxidation or other detrimental degradation. The weak emission of **PTM** with ϕ of 0.7% peaks at 609 nm (in tetrachloromethane (CCl_4_)) [40]. The racemic mixture of right- and left-handed propellers can be resolved by chiral high pressure liquid chromatography (HPLC) into the *P*- and *M*-enantiomers, respectively [40–41]. However, due to the low racemization barrier of only about 22 kcal mol^−1^, the enantiomers racemize within minutes at room temperature. **TTM** is synthesized with slightly improved ϕ of 2% and a blue-shifted emission compared to **PTM** with a maximum photoluminescence wavelength λ_em_ = 569 nm (in CCl_4_) [40,42]. The racemization barrier remains almost identical at 21.1 kcal mol^−1^. Both **PTM** and **TTM** exhibit circularly polarized emission (CPL) for the time that they remain in their enantiopure state. The *g*_lum_ as a measure for the strength of CPL is of order 8 × 10^−4^ for **PTM** and 5 × 10^−4^ for **TTM**.

#### Mixed halide triarylmethyl radicals

Substitution of all *para*-positions in **PTM** with iodine atoms yields the **3I-PTM** radical with a red-shifted emission compared to **PTM**, peaking at 625 nm in tetrahydrofuran (THF) (see Figure 1a). Unfortunately, the photostability of *para*-iodinated radicals is too low to record ϕ in solution [43]. However, when the **3I-PTM** radical is incorporated into a crystalline matrix of the closed-shell **3I-PTM-H** compound, the radical is stabilized and a remarkably high ϕ of 91% can be recorded. This high value is explained by a reduction of the non-radiative pathways by inclusion of the **3I-PTM** into the rigid **3I-PTM-H** crystal, and a charge transfer (CT) from the high bandgap **3I-PTM-H** matrix to the **3I-PTM** radical, which is further supported by the heavy atom effect of iodine, that also allows intersystem crossing (ISC) between triplet states in the matrix and the emissive doublet states of **3I-PTM**. Interestingly, functionalization of all three *para*-positions in **TTM** with chalcogens in the form of aryloxy- and aryl thioethers leads to improved photostability (see Figure 1a). While the triaryl oxyether-**TTM** does not enhance the ϕ, triaryl thioether-**TTM** exhibits a ϕ of up to 37.5% (in cyclohexane). Due to the stronger electron donating effect of the chalcogens compared to halogens, the emission is shifted to lower energies with λ_em_ = 625 nm for the triaryl oxyether-**TTM** and λ_em_ = 655 nm for the triaryl thioether-**TTM** (in dichloromethane (DCM)) [44]. The stronger bathochromic shift and the loss of vibrational features in the photoluminescence spectrum point towards a CT excited state in the triaryl thioether-**TTM**, which could explain the strong increase in ϕ; however, no further information is given to support this claim.

**Figure 2:**
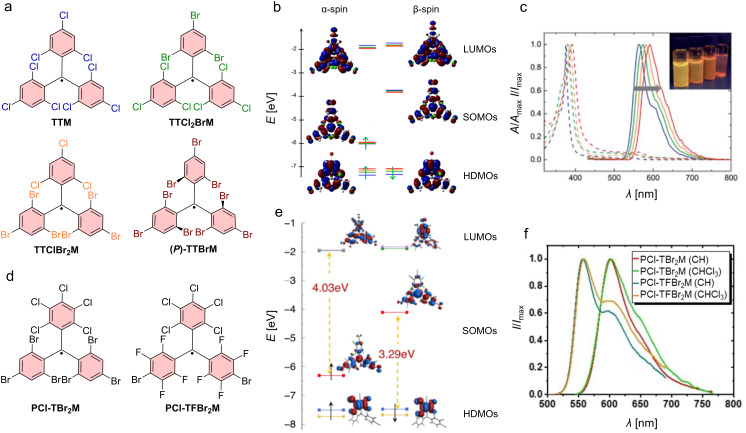
Mixed halide tri- and perhalogenated triphenylmethyl radicals: a) Molecular structures of homo- and mixed chloride/bromide tris(2,4,6-trihalophenyl)methyl radicals (homo-halo **TTM** in blue and **TTBrM** in red (here the stable *P*-enantiomer is shown) and the mixed halo **TTCl_2_BrM** in green and **TTClBr_2_M** in orange). b) Energy diagram of the frontier orbitals of all four radicals shown in a, the color of the energy level corresponds to the above indicated radicals. c) Systematic shift in the UV–vis absorption (dashed line) and photoluminescence (solid line) spectra. The line color of the spectra corresponds to the above indicated color code. d) More mixed halide triarylmethyl radicals with a perchlorinated phenyl ring and two 2,4,6-tribromopenyl rings (**PCl-TBr_2_M**) or two tetrafluorinated-monobrominated rings (**PCl-TFBr_2_M**). e) Energy diagram of the frontier orbitals of **PCl-TFBr_2_M** (the energy levels are calculated using B3LYP/6-31+G** and ωB97X-D/6-31+G** represented by the different colors). The frontier orbitals indicate that the pentachlorinated ring might act as an electron-donor moiety. f) Fluorescence spectra of **PCl-TBr_2_M** and **PCl-TFBr_2_M** in cyclohexane (CH) and chloroform (CHCl_3_). Figure 2b and Figure 2c were adapted from [50] (“Mixed-halide triphenylmethyl radicals for site-selective functionalization and polymerization“, © 2021 L. Chen et al., published by RSC, distributed under the terms of the Creative Commons Attribution-NonCommercial 3.0 Unported Licence, https://creativecommons.org/licenses/by-nc/3.0/). This content is not subject to CC BY 4.0. Figure 2e and Figure 2f were adapted from [52], D. Mesto et al., “Tuning the Electro-Optical Properties of Mixed-Halide Trityl Radicals Bearing para-Brominated Positions through Halogen Substitution”, *Eur. J. Org. Chem.*, with permission from John Wiley and Sons. Copyright © 2022 WILEY‐VCH GmbH. This content is not subject to CC BY 4.0.

While substitution of all *para*-positions with hetero-halogens or chalcogens does not induce a different symmetry in the ground state of the molecule, substitution of only one of the *para*-chlorines of **PTM** with bromine or iodine should break the symmetry; however, no effect on the absorption spectra and especially on the lower energy |D_1_⟩ transition has been reported [45]. Interestingly, substitution of one of the *para*-chlorines in **TTM** by iodine (**I-TTM**) has been reported to enable Pd-catalyzed cross-coupling, allowing functionalization with a much broader variety of donors (and even acceptors) than is possible through a radical-mediated nucleophilic aromatic substitution (S_RN_1, see also below) [3,46–47]. The **I-TTM** has a maximum emission at λ_em_ = 578 nm and a ϕ of 3% (in cyclohexane), indicating that the symmetry does not seem to be altered sufficiently to evoke a noticeable improvement of the ϕ [48]. This also holds true for mono-*para*-substitution with boronic acid derivatives, yielding λ_em_ = 580 nm and ϕ of 1–3% (in dichloromethane) [49]. Substituting one or two of the 2,4,6-trichlorinated rings in **TTM** with 2,4,6-tribromophenyl units shifts the photoluminescence systematically towards the red (see Figure 2a–c) [50]. The ϕ also decreases systematically, demonstrating that also more pronounced mixed halogenation does not significantly change the symmetry of the locally excited (LE) state. The tris(2,4,6-tribromophenyl)methyl (**TTBrM**) radical exhibits λ_em_ = 593 nm and ϕ of 0.8% (in dichloromethane-solution, at room temperature) [50–51].

Functionalization of **TTM** in the *para*-position has also been achieved with a pseudo-halide, namely a nitrile group. While the absorption and emission spectra are slightly bathochromically shifted for mono- and bis-*para-*nitrile **TTM** radicals, successive nitrilation increases the ϕ to 4.6% and 7.4% (in cyclohexane) [53]. This improvement is most likely induced by symmetry breaking, resulting in excited states with some charge-transfer character. Moreover, the photostability of such nitrile-bearing **TTM** equivalents is greatly enhanced.

Interestingly, much like **TTM**, **TTBrM** exists also as enantiomeric propellers; however, in **TTBrM** radicals the resolved enantiomers are stable at room temperature, making these molecules interesting as chiral emitters with *g*_lum_ of 7 × 10^−4^, despite their low ϕ (see Figure 2a) [50–51].

Perfluorinated triphenylmethyl radicals have been reported as well; however, neither UV–vis nor photoluminescence data are available [54]. Mixed perhalogenated triphenylmethyl radicals with fluorine, chlorine, and bromine substituents have been synthesized in an attempt to break the symmetry. Here, the molecule consists of a pentalchloro phenyl and two tribromophenyl units (**PCl-TBr_2_M**) [55], or with two *p*-bromotetrafluorophenyl rings (**PCl-TFBr_2_M**) (see Figure 2d–f) [52]. DFT calculations for these mixed per- and trihalophenylmethyl radicals and their orbitals show a change from the *D*_3_ symmetry as observed in **TTM** to a *C*_2_ symmetry in the orbital distribution in the ground state. In spite of this break in symmetry to *C*_2_ – where all relevant transitions will be allowed – an enhancement of the respective absorption bands or an improvement in the ϕ has not been observed. However, one can observe a loss of the vibronic features in the fluorescence signal, indicating that the emission occurs from a CT state, in agreement with the DFT orbital distribution in the ground state geometry (see Figure 2e for different orbital location in HDMO and SOMO). Interestingly, successive bromine substitution decreases the spin–lattice relaxation time *T*_1_ of the radical electron. This is due to increased spin–orbit coupling in the brominated species. By contrast, the phase memory time *T*_m_ becomes longer for successively brominated radicals, visible from the concurrent spectral broadening in electron paramagnetic resonance (EPR). This enables improved homogeneity in quantum phase evolution and superior *T*_m_, rendering brominated trityl radicals interesting for quantum memory applications [55].

#### Symmetry in *N*-heteroaryl-functionalized diphenylmethyl radicals

**Figure 3:**
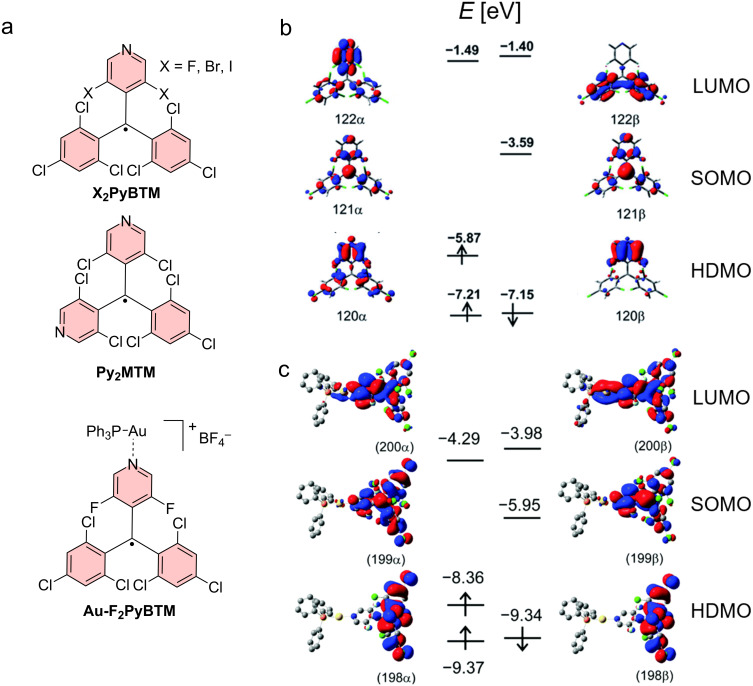
Pyridine-functionalized triarylmethyl radicals. a) Chemical structures of **X_2_PyBTM**, **Py_2_MTM**, and **Au-F_2_PyBTM**. b) Energy diagrams and frontier orbitals of **F_2_PyBTM**, and c) of **Au-F_2_PyBTM**. Figure 3b was adapted with permission of The Royal Society of Chemistry, from [6] (“Highly photostable luminescent open-shell (3,5-dihalo-4-pyridyl)bis(2,4,6-trichlorophenyl)methyl radicals: significant effects of halogen atoms on their photophysical and photochemical properties” by Y. Hattori et al., *RSC Adv.*, vol. 5, issue 79, © 2015); permission conveyed through Copyright Clearance Center, Inc. This content is not subject to CC BY 4.0. Figure 3c was adapted with permission of The Royal Society of Chemistry, from [57] (“Synergistic luminescence enhancement of a pyridyl-substituted triarylmethyl radical based on fluorine substitution and coordination to gold” by Y. Hattori et al., *Chem. Commun.*, vol.52, issue 91, © 2016); permission conveyed through Copyright Clearance Center, Inc. This content is not subject to CC BY 4.0.

Another approach to affect the symmetry in triarylmethyl radicals is by touching the triphenylmethyl scaffold itself, rather than only its substitution pattern. A variety of studies have been concerned with the pyridine-diphenylmethyl (**PyBTM**) motif, which has one of the *para*-carbons replaced by a nitrogen (see Figure 3) [56]. Surprisingly, these molecules display greatly enhanced photostability compared to **PTM** and **TTM**. The *ortho*-positions on the pyridyl ring have been substituted with fluorine, chlorine, and bromine moieties, and it was found that the change in substitution affects the emission color from yellow in case of fluorine, to orange for chlorine, and to red for bromine [6,57]. This shift can be explained by the decreasing electronegativity of the halide substituents. The more electronegative fluorine will reduce the HDMO energy more strongly than the bromine, while the SOMO remains energetically almost unaffected, explaining the higher energy gap with increasing electronegativity.

Moreover, the photostability increases from fluorine to bromine-substituted **PyBTMs**. Higher photostability has only been observed in a bispyridyl-phenylmethyl radical (**Py_2_MTM**) [58]. While **PyBTM** is 70 times more stable than **TTM**, the photostability in **Py_2_MTM** is increased 3000-fold. The nuclear spin of the nitrogen in **PyBTM** and **Py_2_MTM** can couple with the radical electron spin, leading to EPR spectra with hyperfine structure [58].

Effectively, the substitution in *ortho*-position has strong electronic as well as steric effects, allowing to tune the emission properties of such **X_2_PyBTM** molecules [6]. The ϕ is highest for the **F_2_PyBTM** with 6%, **Cl_2_PyBTM** has 3% and **Br_2_PyBTM** 2% [6,57]. The increase in ϕ for the most electronegative substituent might be explained by a somewhat more pronounced CT excited state, due to the stronger electron-withdrawing character of the fluorine substituent. When gold atoms are coordinated to the pyridine nitrogen, a ϕ as high as 8% has been observed [57]. The reason for this increase in ϕ to 8% for **Au-Cl_2_PyBTM** and 20% for **Au-F_2_PyBTM** is unknown, but the increase in the presence of a heavy atom might point at an improved ISC, opening up more pathways for radiative relaxation.

**Figure 4:**
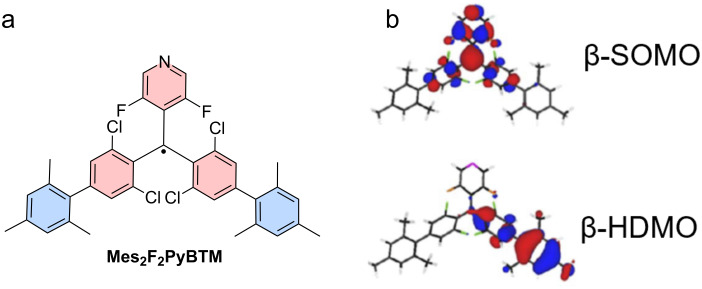
Pyridine-functionalized triarylmethyl radicals. a) Molecular structure of **Mes_2_F_2_PyBTM**, and b) its frontier orbitals. Figure 3b was adapted with permission of The Royal Society of Chemistry, from [59] (“The simplest structure of a stable radical showing high fluorescence efficiency in solution: benzene donors with triarylmethyl radicals” by Y. Hattori et al., *Chem. Sci.*, issue 45, © 2022, distributed under the terms of the Creative Commons Attribution-NonCommercial-NoDerivatives 4.0 International License, https://creativecommons.org/licenses/by-nc-nd/4.0/).); This content is not subject to CC BY 4.0.

When one couples mesityl units as weak donors to the *para*-positions of the two chlorophenyl rings of a **F_2_PyBTM**, the ϕ is improved to 69% (in **Mes_2_F_2_PyBTM**) (see Figure 4) [59]. The DFT studies suggest that there is a clear CT excited state, where the hole will reside on the mesityl and phenyl units (HDMO) and the electron resides on the **PyBTM** core (SOMO) (see Figure 4b).

**Figure 5:**
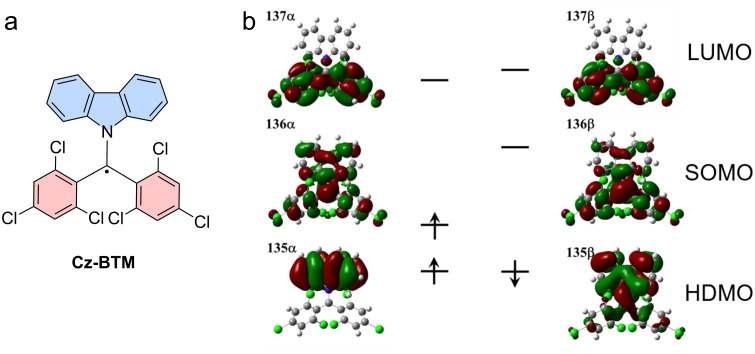
Carbazole functionalized triarylmethyl radical. a) Chemical structure of **Cz-BTM** and b) its energy diagram and frontier orbitals. Figure 5b was adapted with permission from [10], X. Ai et al., “A Stable Room-Temperature Luminescent Biphenylmethyl Radical”, *Angew. Chem., Int. Ed.*, with permission from John Wiley and Sons. Copyright © 2018 WILEY‐VCH Verlag GmbH & Co. KGaA, Weinheim. This content is not subject to CC BY 4.0.

Instead of pyridine also carbazole (**Cz**) units have been coupled to produce *N*-carbazolyl-bis(2,4,6-trichlorophenyl)methyl radicals (**CzBTM**) (see Figure 5a). ϕ is highest at λ_em_ = 697 nm with 2% (in cyclohexane) [10]. DFT calculations display that the **Cz** unit acts as an electron-donor moiety, facilitating a CT excited state with a clear *C*_2_ symmetry (see Figure 5b). Similarly, a biscarbazolylanthracenylmethyl radical has been reported; however, the ϕ has been too low to be determined [60]. Apparently, in these symmetry-broken triarylmethyl radicals there are other non-radiative relaxation pathways at play, reducing the ϕ.

#### Summary: triarylmethyl monoradicals

In conclusion to this section, one has to accept that some of the transitions in *D*_3_-symmetric molecules are forbidden. While the transition to the |D_1_⟩ state is forbidden due to out-of-phase mixing of the degenerate HDMO–SOMO and SOMO–LUMO transitions (as explained by the alternant hydrocarbon rule), the in-phase transition to the |D_2_⟩ state is allowed, as seen in the respective UV–vis absorption spectra of these compounds.

Breaking of the symmetry for the |D_1_⟩ transition by incorporating a donor moiety, leads to enhanced intensity as reflected by growing absorption coefficients ε with increasing donor strength (see Table 1). Replacing one of the phenyl rings for carbazole in triarylmethyl radical leads to much-increased transition dipole moments *M*_|D1⟩_ from ≈1.2 D to 3.0 D, accounting for the much-increased CT character of the |D_1_⟩ state (cf. **TTM** and **Cz-BTM** in Table 1).

**Table 1:** Absorption coefficients ε of the |D1⟩ transition, which increase with increasing charge transfer character of the excited state. Transition dipole moments *M*_|D1⟩_ are determined as part of this study using DFT whereas ε and ϕs are reprinted as reported.

	**PTM** [38]	**TTM** [61]	**TBr_2_Cl_5_M** [52]	**TBr_2_Cl_5_F_8_M** [52]	**PyBTM** [57–58]

ε (× 10^3^ M^−1^ cm^−1^)	1.2 (in CH)	0.84 (in CHCl_3_)	0.94 (in CHCl_3_)	0.54 (in CHCl_3_)	1.0 (in DCM)
*M*_|D1⟩_ (D)	1.2	1.2	1.2	1.36	1.34
ϕ (%)	1	2	1	<1	1–3

	**Br_2_PyBTM** [57]	**F_2_PyBTM** [57]	**Mes_2_F_2_PyBTM** [59]	**Cz-BTM** [10]	**TTM-Cz** [61]

ε (× 10^3^ M^−1^ cm^−1^)	1.0 (in DCM)	0.85 (in DCM)	1.3 (in DCM)	3.06 (in CH)	3.74 (in CHCl_3_)
*M*_|D1⟩_ (D)	1.17	1.46	1.64	3.01	3.32
ϕ (%)	2	6	69	2	88–91

However, for the emission of all triarylmethyl radicals, the symmetry plays less of a role. While the excited state geometry changes to the *C*_2_ symmetry, where all relevant transitions are allowed, the ϕ varies between <1% and 69%.

Therefore, the poor emission performance in triarylmethyl radicals seems to be primarily governed by the CT strength of the excited state. For radicals with more LE contribution to the |D_1_⟩ state, dark quartet states might play a more prominent role in the non-radiative relaxation than currently acknowledged in the literature. The quartet state in triarylmethyl radicals – which is analogous to the dark triplet relaxation pathway in closed-shell emitters – might contribute to non-radiative excited-state relaxation (because of spin selection rules forbidding the direct transition from the quartet to the doublet state). While for **TTM** and **PTM** substituted with weak donors such energetically low-lying quartet (Q_1_) states have been observed in TD-DFT calculations [20,62] and EPR spectroscopy [63], there are no such investigations reported for the pure **TTM** and **PTM** systems. Also, the widespread assumption that the Q_1_ in triarylmethyl radicals is typically higher in energy than the D_1_ state, might not always hold true, as the polarity of the environment, for example solvents or matrices, might shift the respective energy levels. In the future, the tuning and energetic positioning of the quartet states should receive more attention to improve the photoluminescence performance of triarylmethyl radicals. Alternatively, there might be strong vibronic interactions, which could also contribute to non-radiative decay of the excited state in trityl radicals. Depending on the pattern of functionalization, the frequency of such vibrational modes might be shifted, or the vibrations might be suppressed altogether. These non-radiative decay pathways could explain the variable performance of trityl radicals and should be further investigated in the future.

In view of future applications in quantum technology, further effort should be directed at ways to increase coherence time, as was done in the study of increasing bromination of trityl radicals [55]. Moreover, such systems would also benefit from strategies to increase their fluorescence, as this would potentially allow to read out their spin state optically.

### Donor-functionalized triarylmethyl monoradicals

#### Mono-functionalized triarylmethyl radicals

**Figure 6:**
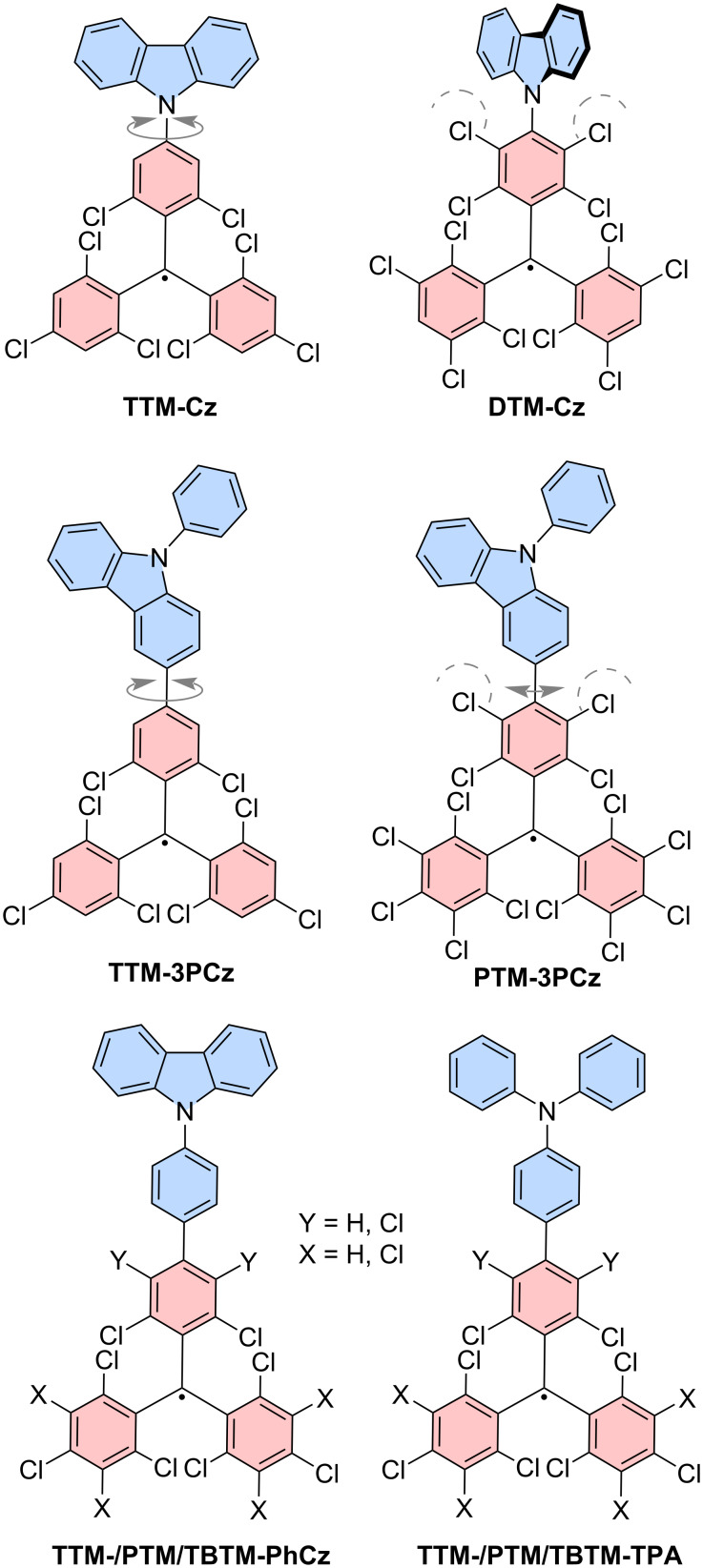
Donor-functionalized triphenylmethyl radicals. Molecular structures of **TTM-Cz**, **DTM-Cz**, **TTM-3PCz**, **PTM-3PCz**, for X,Y = H: **TTM-PhCz** and **TTM-TPA**, for X,Y = Cl: **PTM-PhCz** and **PTM-TPA** and Y = Cl and X = H: **TBTM-PhCz** and **TBTM-TPA**. The dashed lines indicate steric demand of the chlorine substituents. The grey arrows indicate rotational freedom around the respective bond.

Functionalization of the triphenylmethyl radical with electron-donating **Cz**, has been reported long before the above discussed **Cz-BTM** (see Figure 6). In fact, **TTM-Cz** is the first donor-functionalized triarylmethyl radical that has been reported and immediately showed very high ϕ between 88–91%, with λ_em_ = 628 nm (in cyclohexane, the original report only claimed 53%, which was determined using a relative method [61,64]) [65–66]. Like in the heteroaryl-**BTM** radicals, the donor moiety produces a CT excited state, where the hole (h^+^) resides on the **Cz** unit and the electron (e^−^) resides on the **TTM** unit. The previously discussed low energy absorption becomes somewhat more pronounced in **TTM-Cz**, which is sensible as **TTM-Cz** exhibits a *C*_2_ geometry in the ground state, where all relevant transitions will be allowed. However, CT transitions typically exhibit a low oscillator strength and therefore it is not surprising that the corresponding absorption is not strongly enhanced by attachment of the **Cz** unit [19,61].

The same appears to be true for donor functionalization of **Cz-BTM**. Attachment of an acridine-derived donor to the *para*-position of one of the phenyl units in the **BTM** subunit, boosts the ϕ to 55% (in toluene) [67]. Quantum chemical calculations corroborate that the CT excited state is much more pronounced in acridine functionalized **Cz-BTM** than in pristine **Cz-BTM**, with a charge separation distance in the excited state of 0.68 nm versus 0.21 nm, respectively.

Quantum chemical investigations have also been employed to understand the strong emission observed experimentally in **TTM-Cz**. One explanation is based on intensity borrowing from higher excited states. While first hypotheses suggested a vibronic coupling between the D_1_ and D_2_ states on the **TTM** moiety [19], recent calculations propose vibronic intensity borrowing from higher excited states as high as D_7_, which represent LE states with high oscillator strengths that are typically in resonance with the LE D_2_ state [68]. However, elsewhere it was found that the oscillator strength of such resonant higher lying states may be insufficient for substantial intensity borrowing that could explain the high ϕ [21].

The role of Q_1_ states as a dark deactivating pathway has been investigated by DFT analysis. The position of the spin-quartet is in part dictated by the triplet state of the donor moiety. For example, strong donors like benzocarbazole exhibit low lying triplet states, which in combination with the **TTM** unit lead to the Q_1_ state being reduced in energy below the D_2_ states [20,69]. Here, the Q_1_ state remains energetically above the D_1_ state so that it does not seem to affect the ϕ, but one might consider other donor geometries and substitution patterns, where the Q_1_ energy is reduced even further, so that it interferes with the excited-state relaxation.

Quantum chemical investigations have also brought forth that the dihedral angle between the **TTM** radical plane and the carbazole donor increases when exciting the molecule from the D_0_ ground state to the excited D_1_ state. In the D_1_ state the **Cz** is aligned almost perpendicular to the **TTM** plane, which will stabilize the charge transfer state [37]. This substantial conformational change between the ground and excited states could also be responsible for the comparably long fluorescence lifetimes τ, which are in the range of 30–50 ns, which is long even for CT excited states [68]. The steric demand as well as electronic effects will cause the **Cz**-unit to twist out of conjugation almost completely with the **TTM** radical core plane, leading to marginal orbital overlap between the D_0_ and D_1_ states. The reason for this increase in fluorescence lifetime τ is not completely understood; however, if the emission involves a CT state and the orbital overlap between D_1_ and D_0_ is small, then this could lead to stable D_1_ and long τ [69–71].

Interestingly, the **Cz**-functionalized tris(2,3,5,6-tetrachlorophenyl)methyl radical (**DTM-Cz**) has been synthesized, in which the chlorines in the *meta*-positions of the phenyl ring sterically arrest the **Cz** unit in a position perpendicular to the **DTM** plane (see Figure 6) [47]. This geometry resembles the excited state in **TTM-Cz**, while a change in the dihedral angle between donor and radical plane is almost impossible – meaning that the dihedral angle is almost identical in the ground and excited state geometries. Surprisingly, this leads to a decrease in the ϕ to 2%, while the emission wavelength is at 700 nm (in cyclohexane). Moreover, DFT calculations display that the **Cz**-donor moiety is not involved in the frontier orbitals and the excited state resembles an LE state, which is almost exclusively located on the triphenylmethyl fragment, which might explain the similar ϕ to **PTM** (0.7%) and **TTM** (2%). This suggests that sufficient overlap between the D_0_ and D_1_ charge transfer states is required to achieve strong ϕ. Interestingly, when **3PCz** is connected to **PTM** (**PTM-3PCz**) the ϕ is enhanced to 57%, as opposed to **TTM-3PCz** with a ϕ of 27% (see Table 2) [72–73]. Here, the slight restriction in rotation around the bond between the radical plane and donor seems to improve the radiative relaxation yield. The same is observed in triphenylamine (**TPA**) and phenylcarbazol (**PhCz**) where the ϕ is improved when connected to **PTM** rather than the **TTM** radical moiety. These results point at the involvement of the orbital overlap integral between the D_0_ ground state and the D_1_ excited state playing a role in determining the ϕ of donor-functionalized triarylmethyl radicals.

To provide evidence that this effect is not due to the changed electron accepting strength, **TTM** radicals with additional chlorination only at the phenyl ring, to which the donor moiety is connected have been synthesized. Also here, a strong increase of the ϕ compared to the donor-functionalized **TTM** radicals has been observed (see Table 2). Analysis of the Huang-Rhys factors delivered that the *meta*-chlorinated phenyl units do indeed restrict the rotation of the donor. Moreover, the reduction of the LE character of the D_1_-excited state and a reduction of coupled vibrational modes in the regime from 1000–2000 cm^−1^ seem to decrease the non-radiative pathways, while the rates for emission remain almost unchanged between the donor-functionalized **TBTM** and **TTM** series [73].

**Table 2:** Quantum yields ϕ for three chlorinated trityl radical moieties and four different donors, measured in cyclohexane solution.

ϕ	**Cz**	**3PCz**	**PhCz**	**TPA**

**TTM**	88–91%	27%	4%	12%
**PTM**	2%	57%	44%	26%
**TBTM**		59%	16%	43%

This effect has also been investigated by excited state vibrational spectroscopy, where (among other compounds) **TTM-TPA** and **TTM-3PCz** have been investigated [74]. The donor-functionalized trityl molecules exhibit only few vibrational modes in the above mentioned spectral range, leading to relatively high ϕ at long wavelengths (≈800 nm). These molecules seem to overcome the typical restriction of the energy gap rule, where the ϕ of conventional fluorophores deteriorates in the near-infrared region.

**Figure 7:**
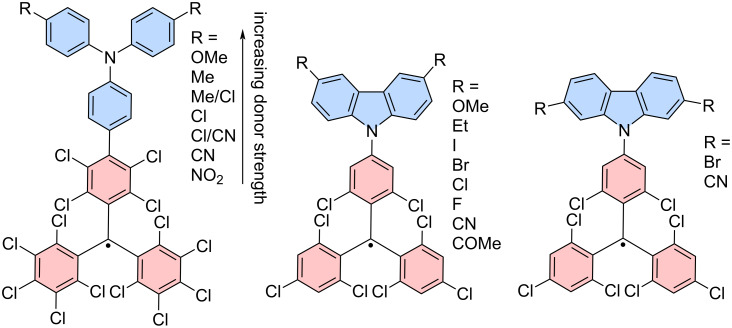
Tuning of the donor strength. Functionalization with electron-donating and electron-withdrawing groups on **TPA** and **Cz** donors. The electron-donating and -withdrawing units are organized in accordance with their effect for an increasing donor strength (see arrow) [21,64–65,75].

Moreover, the donor strength plays an important role for the ϕ. A slight adjustment of the donor strength of **TPA** has been investigated in **PTM-TPA** by introducing electron-donating and electron-withdrawing units on the *para*-position of the free phenyl rings in **TPA** (see Figure 7). For chlorine substituents an optimized ϕ of 38% with λ_em_ = 763 nm could be achieved (in cyclohexane) [71]. Units that are more electron-donating increase the donor strength of **TPA**, while electron-withdrawing units will reduce the donor strength of **TPA**, both effects lead to decreasing ϕ. This observation has been explained by the potential of the **TPA** unit to stabilize the positive charge that would reside here after excitation and formation of the CT state [71]. In a similar fashion also electron-donating and withdrawing units on **Cz**-donors in **TTM-Cz** have been screened; however, the ϕ could not be improved beyond that of **TTM-Cz** (see Figure 7) [21,64–65,75].

**Figure 8:**
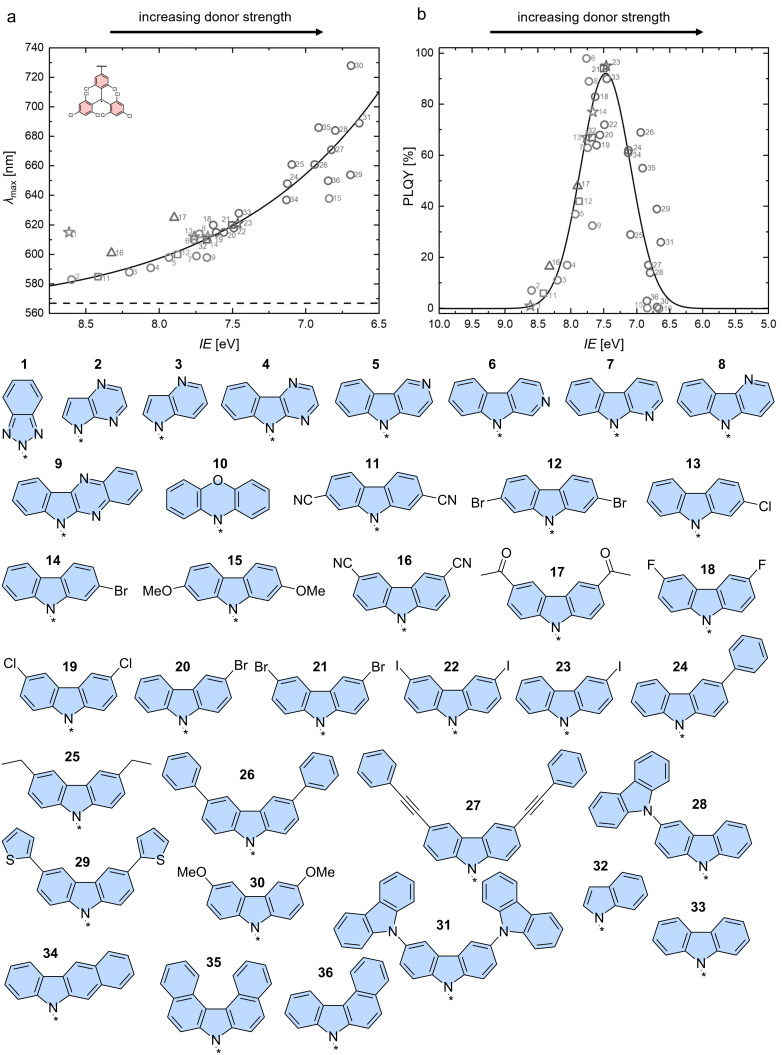
Tuning of the donor strength, by varying the **Cz**-derived donor (**1**–**36**) on a **TTM** radical fragment. a) The donor strength is inversely related to the ionization energy (*IE*) and the maximum photoluminescence wavelength increases with increasing donor strength. The emission wavelength of **TTM** is given as a reference represented by the dashed line. b) ϕ as a function of the donor strength (respectively *IE*) shows a decrease in the ϕ for strong and weak donors and a sweet spot for medium strength donors with high ϕ. Figure 8a,b were adapted from [63] (© 2024 M. E. Arnold et al., published by Wiley-VCH GmbH, distributed under the terms of the Creative Commons Attribution-NonCommercial-NoDerivatives 4.0 International License, https://creativecommons.org/licenses/by-nc-nd/4.0/). This content is not subject to CC BY 4.0.

An expanded meta-analysis of unprecedented and reported **TTM**-based radicals functionalized with **Cz**-derivatives and other *N*-coupled donors has provided further insight into the effect of the donor strength on the emission wavelength maximum λ_em_ and the ϕ (see Figure 8) [63].

**Figure 9:**
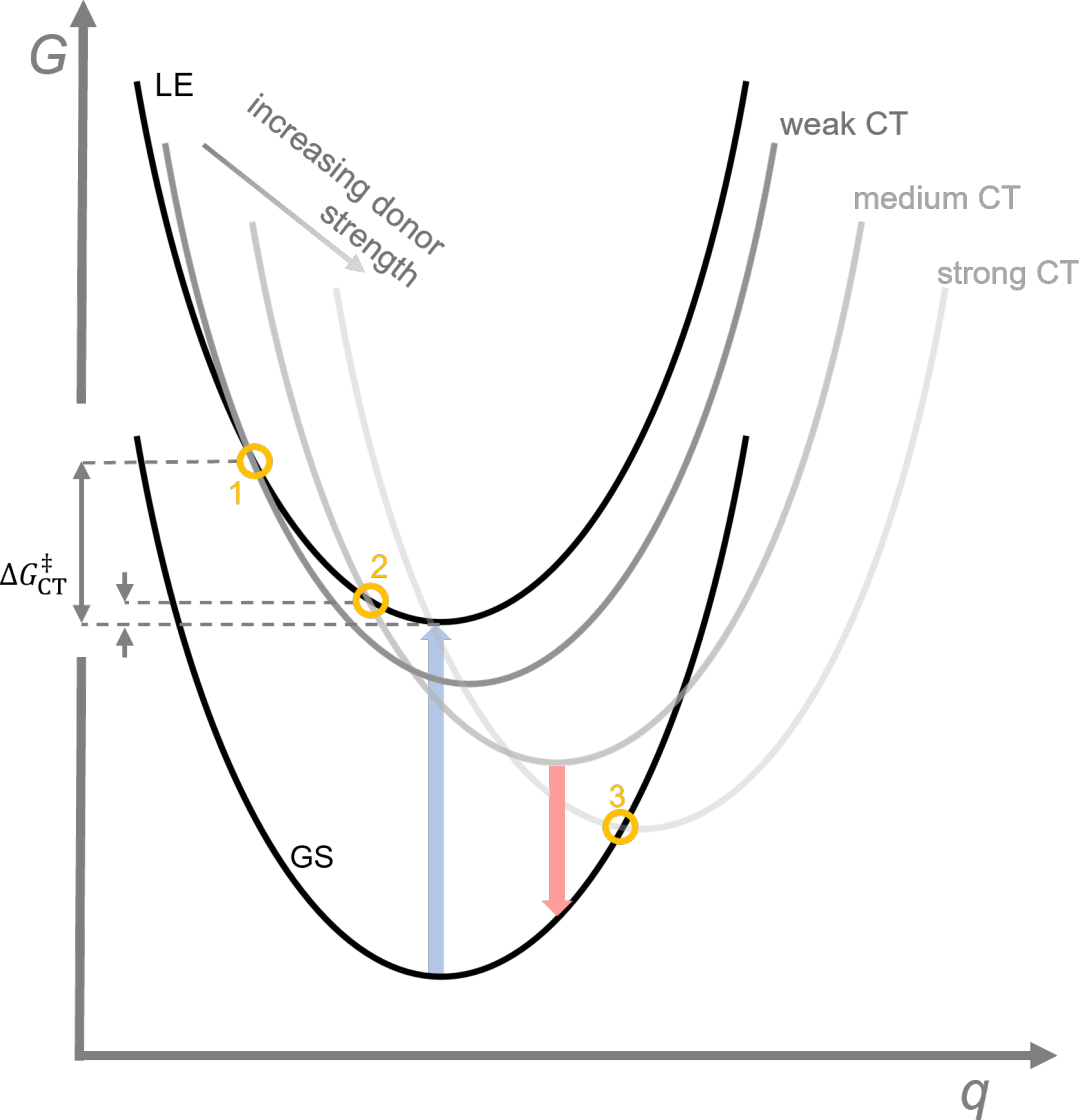
Three-state model and Marcus theory: *q* is the charge transfer coordinate and *G* the free energy. Ground state (GS), locally excited (LE) state and charge transfer (CT) states evoked by weak to strong donors. Excitation (blue) ensues adiabatically into the excited LE state. Depending on the donor strength, the activation energy to cross over to the CT states is high (point 1), low (point 2) or barrierless (for strong donors). In case of strong donors the CT state might exhibit a conical intersection (point 3), which allows non-radiative relaxation into the GS instead of radiative relaxation (red).

In the meta-analysis the donor strength is approximated by the ionization energy (*IE*), which has been determined using DFT calculations. Plotting of the maximum emission wavelength and the ϕ versus the calculated *IE*s produces a systematic increase for λ_em_ and a bell-shaped distribution for ϕ. The bathochromic shift in λ_em_ has previously been described to result from the better stabilization of the CT excited state for stronger donors, and therefore a stabilization of the highest occupied molecular orbital (HOMO) when only considering the acceptor moiety (see also reduction in energy from weak to strong CT in Figure 9) [62,71]. The bell shape of the ϕ dependency on the donor strength could be explained by employing a three-state model, in which one assumes that the excitation of the donor-functionalized **TTM** radical occurs adiabatically into an LE state, followed by relaxation into the CT D_1_ state, from where emission back to the D_0_ ground state may occur.

Weak donors will exhibit a high energy transition state to crossover from the excited LE to the CT state (large Δ*G*_CT_^‡^, point 1 in Figure 9). In absence of any excess energy, the LE will relax non-radiatively to the ground state without crossing over to the CT state. The fact that excited LE states relax non-radiatively has been discussed above for **TTM**, **PTM**, as well as for **DTM-Cz**, all of them featuring low ϕs. Interestingly, for the very weak benzotriazole unit a quartet state could be identified after excitation, hinting at the type of non-radiative decay pathways in these molecules [63]. For medium strong donors the Gibbs energy of activation Δ*G*_CT_^‡^ for crossing over into the CT state is low, and emission can occur from here with high ϕ (see point 2 in Figure 9). For strong donors, Δ*G*_CT_^‡^ is close to 0 allowing almost barrier-free transition into the CT state. However, here a conical intersection with the ground state can occur, which enables non-radiative relaxation to the ground state and therefore low ϕ (see point 3 in Figure 9).

**Figure 10:**
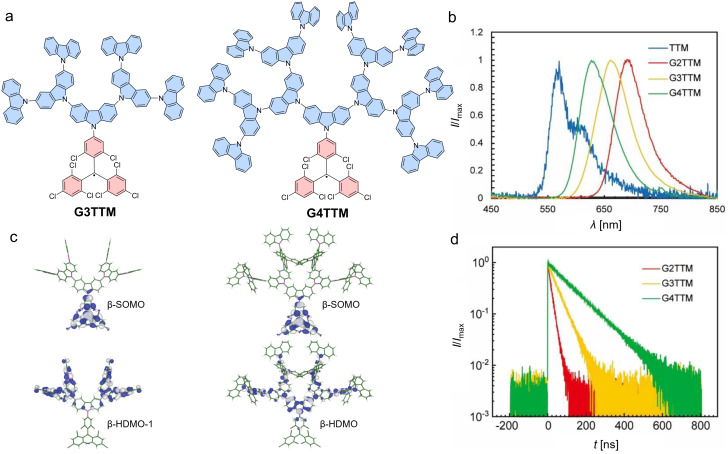
Dendronized carbazole donors on **TTM** radicals. a) Molecular structures of **G3TTM** and **G4TTM**. b) Photoluminescence spectra of **TTM** and **G2TTM** to **G4TTM**. c) DFT calculations of the HDMOs and LUMOs to estimate and visualize the electron–electron Coulomb interactions on the dendronized donors. d) Fluorescence decay spectra, showing the extension in lifetime with growing generations. Figure 10b–d was adapted with permission from [76], R. Xiaotian et al., “Carbazole-Dendronized Luminescent Radicals”, *Angew. Chem., Int. Ed.*, with permission from John Wiley and Sons. Copyright © 2023 Wiley-VCH GmbH. This content is not subject to CC BY 4.0.

This simple three-state model can explain a large variety of different donor–acceptor radical systems; however, it breaks down for very large donor systems. In a dendronized carbazole donor, where the **Cz** units are coupled to the *para*-positions with respect to the *N*-position (first generation: **TTM-Cz**, second generation: **TTM**-tercarbazole (**G2TTM**), third generation: hepta-carbazole-functionalized **TTM** (**G3TTM**), fourth generation: pentadeca-carbazole-functionalized **TTM** (**G4TTM**)) (see Figure 10) [76]. Typically, one would expect the donor strength to increase with extended electron donation throughout the dendronized carbazole donor; however, this is only true for **G2TTM**, which is further red-shifted and reduced in ϕ compared to **TTM-Cz** (cf. compounds **31** and **33** in Figure 8a and b). Higher generations (**G3TTM**, **G4TTM**) exhibit hypsochromically shifted emission maxima compared to the **G2TTM** and increased ϕ (52% and 63% respectively (in cyclohexane)) (see Figure 10). This effect can be explained by recognizing that the donor dendron is large and its inertia prevents fast conformational changes between the D_0_ and D_1_ states. This inertia is also reflected in the increasing fluorescence lifetimes, which increase from τ = 17.3 ns for **G2TTM**, to τ = 48.8 ns for **T3TTM** and τ = 120.0 ns for **G4TTM**. Moreover, the counterintuitive effect of an increasing D_0_ to D_1_ energy gap and ϕ with growing donor strength (from **G2TTM** to **G4TTM**) has been explained by a reduction of non-radiative relaxation processes, due to the decrease of the electron–electron repulsion in the occupied dendron orbitals.

**Figure 11:**
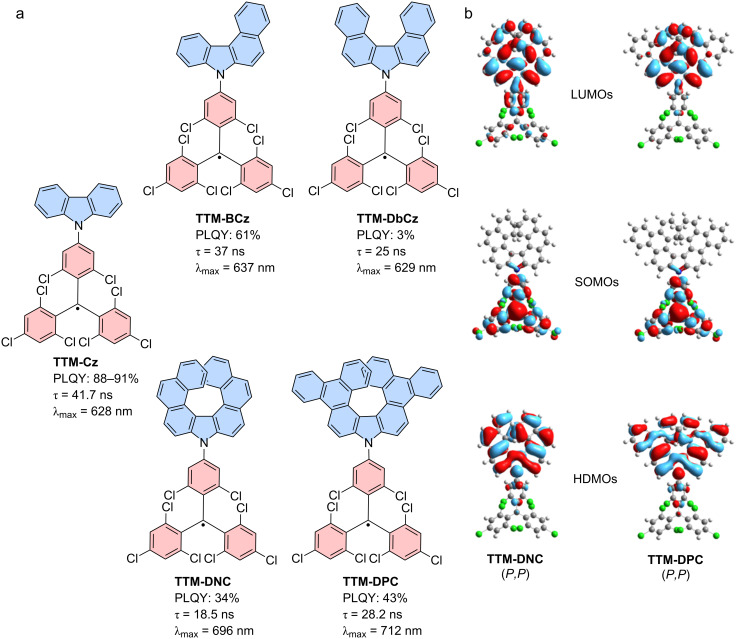
Electronic extension of the **Cz** donor. a) Molecular structures and optoelectronic properties of **TTM-Cz**, **TTM-BCz**, **TTM-DbCz**, and the chiral **TTM-DNC** and **TTM-DPC**, ordered for increasing level of extension. b) Frontier molecular orbitals of the chiral **TTM-DNC** and **TTM-DPC**, the β-spin orbitals for the *P*,*P*-stereoisomers are shown. LUMOs were calculated as part of this review, whereas HDMOs and SOMOs in Figure 11b were adapted from [66] (© 2023 M. Gross et al., published by Wiley-VCH GmbH, distributed under the terms of the Creative Commons Attribution-NonCommercial-NoDerivatives 4.0 International License, https://creativecommons.org/licenses/by-nc-nd/4.0/). This content is not subject to CC BY 4.0.

In a similar fashion, a simple extension of the **Cz** donor structure to benzocarbazole (**BCz**) and dibenzocarbazole (**DbCz**) follows the trend of the above described three-state model (see Figure 11). However, further extension to aza[7]helicenes also leads to contradiction with the model (cf. compounds **35** and **36** in Figure 8a and b). The ϕs of **TTM-BCz** and **TTM-DbCz** decrease in accordance with the increasing strength of the respective donors to 61% and 3%, respectively (in cyclohexane) [77]. By contrast, further extension to **TTM-DNC** and **TTM-DPC** leads to increased ϕs despite bathochromic shifting of the emission wavelength. Here, the strain in the donor molecule might lead to reduced electron–electron repulsion in the extended systems improving the emission characteristics [66].

#### Circularly polarized photoluminescence

The **TTM-DNC** and **TTM-DPC** with their helical donors are chiral and can be separated for the axial chirality of the helicene donor unit (see Figure 11). Chiral chromatography provides access to all four stereoisomers, as both, the helicene and the **TTM** unit, are chiral (as discussed above). However, the **TTM** propellers racemize quickly so that effectively one receives the diastereomers with the respective helicene chirality, and a racemic mixture of **TTM** propellers. Surprisingly, only the **TTM-DNC** showed acceptable CPL with *g*_lum_ = 5 ×10^−4^, whereas the **TTM-DPC**, despite its respectable ϕ, did not exhibit appreciable CPL. While the *g*_lum_ is not higher than for the enantiomerically resolved **TTM** propellers, the **TTM-DNC** diastereomers are stable and in contrast to the **TTM** propellers, they do not racemize further. Moreover, the good ϕ leads to a respectable CPL brightness *B*_CPL_ = 0.25 M^−1^cm^−1^, compared to 0.0007 M^−1^cm^−1^ for **TTM** [66].

Other examples of introducing fixed chiral units beyond the racemizing **TTM** propeller yielded *g*_lum_ as high as 1.51 × 10^−3^, for chiral emitters at 5 wt % dispersed in a matrix of polymethylmethacrylate (**PMMA**). Unfortunately, these pillarene-bridged **TTM** radicals with a **TPA** donor did not exhibit CPL in solution [78]. This report signifies that CPL is more easily achieved in the solid phase, where even racemic mixtures of the interconverting **PTM-PhCz**, **TTM-3PCz**, and **TTM-Cz** propellers can exhibit CPL, when doped into a **PMMA** together with a chiral gelator or when in presence of a chiral (liquid crystalline) mesogen. In these cases, *g*_lum_ in the range of 10^−3^ and 4 × 10^−2^ was obtained respectively [79]. In the latter case, alternation of an electric field would realign the mesogen allowing for reversible switching between CPL and depolarized emission.

#### Multi-donor-functionalized triarylmethyl radicals

Before iodinated **TTM** derivatives became available – enabling mild reaction conditions for precise C–C and C–N cross-coupling reactions only at the site of the iodine – donors were attached to **TTM** by radical-mediated nucleophilic aromatic substitution S_RN_1. The leaving group is the *para*-chlorine atom, of which a **TTM** molecule has three. It is therefore less than surprising that during this S_RN_1 the desired mono-substituted donor-functionalized **TTM** is obtained next to substantial amounts of the bi- and tri-functionalized units. In case of S_RN_1 with **Cz** as a donor, the **TTM-Cz**, **TTM-Cz_2_**, and **TTM-Cz_3_** are obtained. **TTM-Cz_3_** exhibits *D*_3_ symmetry, for which we have previously discussed that certain transitions are forbidden. However, this does not reflect in the ϕs, which for the **TTM-Cz_3_** has been determined to be 52% whereas **TTM-Cz_2_** has delivered 54% and **TTM-Cz** has been determined to be 53% (while more recent studies have produced ϕs between 88–91%) [3]. By contrast, the maximum emission wavelength λ_em_ is not constant and shifts bathochromically from λ_em_ = 628 nm, 651 nm, to 654 nm for increasing donor functionalization (all reported in cyclohexane). Similar results have been obtained for a series of **TTM-3PCz_2_** and **TTM-3PCz_3_** [80]. These data suggest that the ground state symmetry does not play a dominant role here and in fact natural transition orbital (NTO) calculations of a related tris(2,7-dinitrilecarbazole)-**TTM** (**TTM-CzCN_3_**) in its excited state geometry show that the excited state reduces to a *D*_2_ symmetry, where all relevant transitions are symmetry allowed [21]. The nitrile units are electron withdrawing, therefore reducing the donor strength of the **Cz** unit and the ϕ is augmented to 76%, compared to **TTM-Cz_3_**. **TTM-CzCN_3_** shows a clear CT state in the D_1_ geometry, with quite some orbital overlap between the hole (residing on two of the dinitrile-**Cz** units) and the electron (residing on the **TTM** moiety) across the central methyl unit. Such clearly symmetry broken CT states have also been discussed for tri(phenylethynyl)-substituted **PTM** radicals [81] and trimesityl-functionalized **TTM** (**TTM-Mes_3_**) radicals [82–83]. The **TTM-Mes_3_** is of high interest as the mesityl unit is an extremely weak donor (and **TTM-Mes_1_** has only a ϕ of 1% in toluene) and will not by itself induce a strong charge transfer as potent donors like **Cz** do. In **DTM-Cz**, the rotationally restricted **Cz** produces such a CT state even in its ground state, in solvents of high polarity [47]. However, upon excitation of **TTM-Mes_3_** one of the mesityl groups twists further out of the plane of the **TTM**, while the other two units acquire a more planarized structure with the central **TTM** unit. These DFT results show that also **TTM-Mes_3_** performs symmetry breaking upon excitation, which leads to a substantial ϕ of 23% (in toluene) [82].

#### Summary: donor-functionalized triarylmethyl monoradicals

Donor functionalization of trityl radicals induces a CT excited state, which lifts any symmetrical constraints on the emission pathway. However, depending on the strength of the donor, the energies of the states (especially SOMO and LUMO) will be shifted, which can lead to detrimental relaxation pathways through dark quartet states or non-radiative rotational and vibronic relaxation. Typically, the exited-state geometry of donor-functionalized trityl is different from the ground state with the donor unit being twisted into a more or less perpendicular arrangement to the trityl plane. This structural change between excited and ground state entails relatively long fluorescence lifetimes. Future research should be directed towards the challenge of reaching high ϕ, while reducing τ. To date, there is no organic laser reported that employs organic radicals as a gain medium – the reason for this might lie in this discrepancy. For an organic laser one would require high ϕ but short τ.

### Diradicals based on the triarylmethyl motif

Molecules that have two unpaired electrons are coined *diradicals* [84] and in some more specific cases *biradicals* [85], in which the two electrons act nearly independent of each other. *Diradicals* that do exhibit some degree of delocalization and combination of the radical electrons can also be called *diradicaloids*. Since we discuss a variety of different molecules with two unpaired electrons, we will use the more general nomenclature of *diradicals* here – accepting that some of the described molecules would fall under the more specific and accurate terms *biradical* or *diradicaloid*. *Diradical* is the most widely preferred termination in the community, when the general concept and class of molecules with two unpaired electrons is discussed.

**Figure 12:**
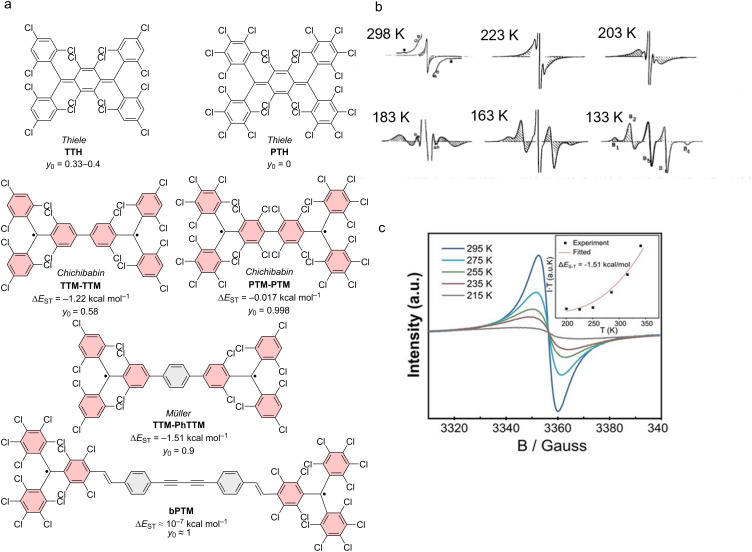
Kekulé diradicals: a) hexadeca- and perchlorinated Thiele (**TTH**, **PTH**), Chichibabin (**TTM-TTM**, **PTM-PTM**), and Müller (**TTM-PhTTM**) diradicals alongside a further extended **bPTM** diradical. b) Variable temperature (vt)-EPR spectra of **PTM-PTM** c) vt-EPR of **TTM-PhTTM**. The inset shows the Bleaney-Bowers fit allowing the determination of Δ*E*_ST_. Figure 12b was adapted with permission from [95], Copyright 1994 American Chemical Society. This content is not subject to CC BY 4.0. Figure 12c was reproduced with permission from [96], A. Abdurahman et al., “A Highly Stable Organic Luminescent Diradical”, *Angew. Chem., Int. Ed.*, with permission from John Wiley and Sons. Copyright © 2023 WILEY‐VCH GmbH. This content is not subject to CC BY 4.0.

**Figure 13:**
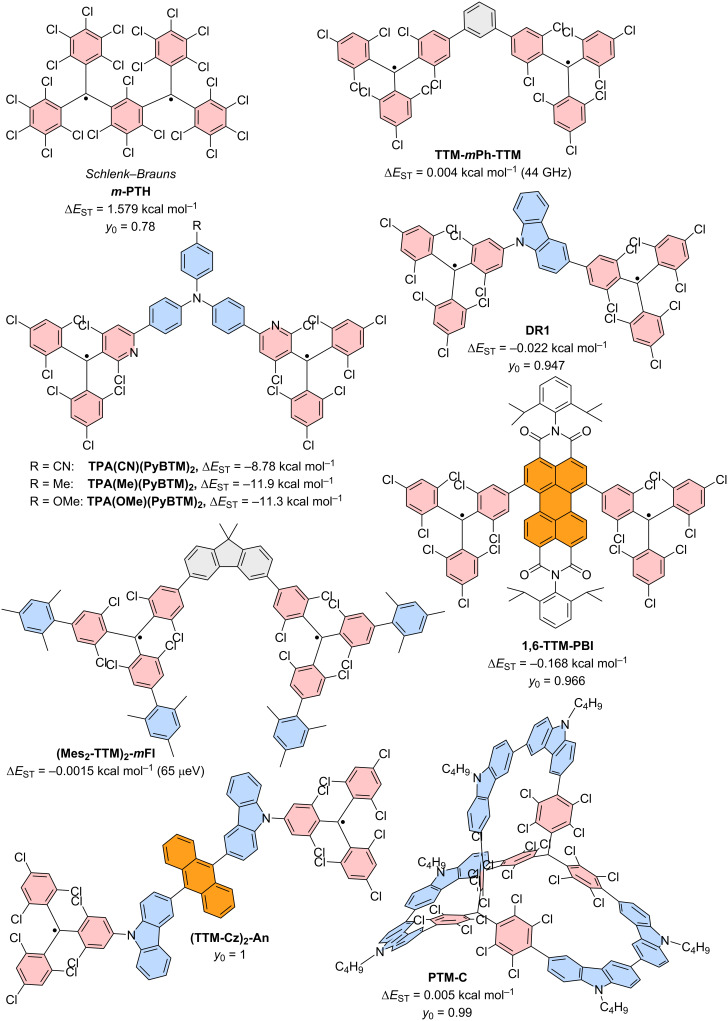
Non-Kekulé diradicals: perchlorinated Schlenk–Brauns radical (***m*-PTH**), *meta*-coupled **TTM** radicals in **TTM-*m*Ph-TTM**, *meta*-fluorene bridged, mesityl functionalized **TTM** diradical **(Mes2-TTM)_2_-*m*Fl**, perylene bisimide-bridged **TTM**-based diradical **1,6-TTM-PBI**, a series of triphenylamine-bridged **PyBTM**-derived diradicals **TPA(PyBTM)_2_** with different electron directing groups, a carbazole-bridged bis-**TTM** (**DR1**), an anthracene-bridged bis-**TTM-Cz** (**TTM-Cz)_2_-An** and a **PTM** diradical derived cage-molecule with three dicarbazole bridges **PTM-C**.

Combination of two trityl motifs to produce diradicals, is a synthetic challenge that is almost as old as the realization of the triarylmethyl radical. Like the triarylmethyl radical or Gomberg’s radical [31], the resulting diradicals typically carry the name of their discoverers. Coupling of two trityl radicals through their *para*-positions results in the Chichibabin hydrocarbon (e.g., **TTM**-**TTM**, **PTM-PTM**) [86]. If an additional phenyl ring is incorporated between the trityl units, we obtain Müller’s hydrocarbon (**TTM-PhTTM**) [87]. When two of the trityl radicals overlap in one ring and the radical electrons can delocalize in a quinodal structure because of the *para*-linkage, these molecules are termed Thiele’s hydrocarbons (e.g., **TTH**, **PTH**) (see Figure 12) [88]. These molecules, in which the electrons can be formally delocalized are termed Kekulé diradicals (diradicaloids). By contrast, if the methylene radicals are connected through the *meta*-positions of the central phenyl ring, Schlenk-Brauns (***m*-PTH**) diradicals are obtained where no Kekulé-conjugation between the radical centers is possible (see Figure 13) [89]. While the *para*-coupled Thiele, Chichibabin, and Müller radicals can acquire a closed-shell quinodal electronic structure, diradicals with broken Kekulé-conjugation exhibit much stronger diradical character. In accordance with the above-described nomenclature, these diradicals are termed non-Kekulé diradicals.

This tendency of diradicals to form a closed-shell electronic configuration can be described using the diradical index *y*_0_, which corresponds to a closed-shell system for *y*_0_ = 0 and a purely open-shell diradical for *y*_0_ = 1. In the open-shell electron configuration, the diradicals can acquire a singlet state with open-shell but antiparallel spins (total electronic spin, *S* = 0) or a triplet ground state with parallel spins and *S* = 1 for the diradical molecule. Whether a molecule has a singlet or triplet ground state can be expressed by the energy difference between the first singlet and first triplet states Δ*E*_ST_. When the energy difference is negative, Δ*E*_ST_ < 0, the diradical exists in a singlet ground state. When the energy difference is positive Δ*E*_ST_ > 0, the diradical has in a triplet ground state. Often the energy difference is very small, so that thermal effects come into play and higher lying electronic states can be populated at room temperature.

In the following, we will discuss such diradicals and we group the molecules in accordance with their type of conjugation.

#### Kekulé-conjugated diradicals

**Figure 14:**
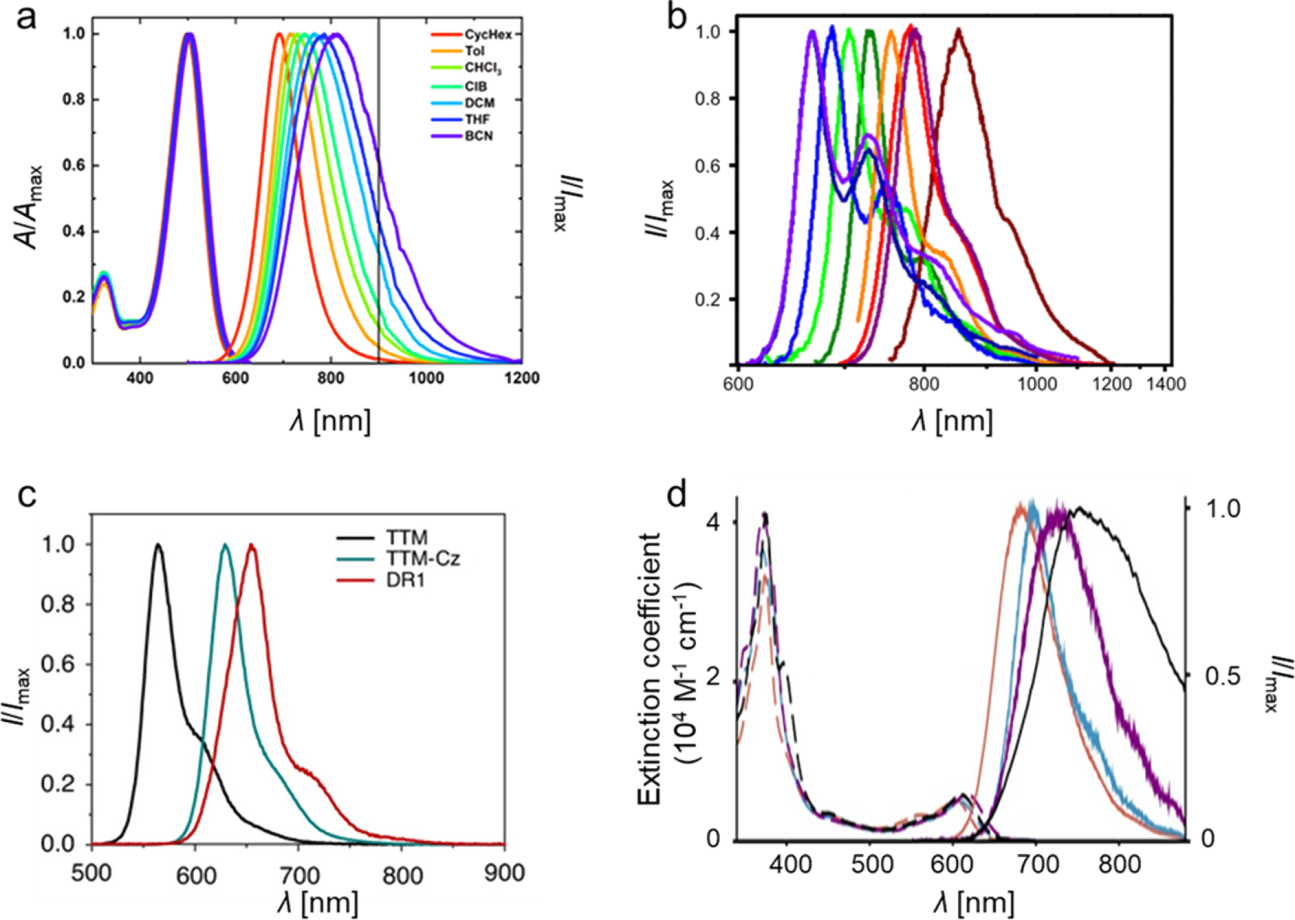
UV–vis absorption and photoluminescence spectra of a) **TTH** in solvents of different polarity, b) diradicals: **TPA(CN)(PyBTM)_2_** (light green), **TPA(Me)(PyBTM)_2_** (orange), **TPA(OMe)(PyBTM)_2_**, triradicals: **TPB(PyBTM)_3_** (purple), **TPA(PyBTM)_3_** (dark green), all recorded in cyclohexane (the other colors belong to monoradicals and other species not important to this review), c) **TTM**, **TTM-Cz**, **DR1** all in cyclohexane, d) extinction and photoluminescence spectra of **TTM-Cz** (red), **TTM-Cz**-anthracene fragment (turquoise), **TTM-Cz** coupled to a phenylanthracene (purple) and **(TTM-Cz)_2_-An** in toluene. Figure 14a was adapted with permission from [92], Copyright 2023 American Chemical Society. This content is not subject to CC BY 4.0. Figure 14b was adapted from [107] (© 2019 Y. Hattori et al., published by Wiley-VCH Verlag GmbH & Co. KGaA, distributed under the terms of the Creative Commons Attribution-NonCommercial 4.0 International License, https://creativecommons.org/licenses/by-nc/4.0/). This content is not subject to CC BY 4.0. Figure 14c was adapted from [108] (© 2020 A. Abdurahman et al., published by Springer Nature, distributed under the terms of the Creative Commons Attribution 4.0 International License, https://creativecommons.org/licenses/by/4.0). Figure 14d was adapted from [109] (© 2023 S. Gorgon et al., published by Springer Nature, distributed under the terms of the Creative Commons Attribution 4.0 International License, https://creativecommons.org/licenses/by/4.0).

Trityl radicals and trityl-derived diradicals have been reported to be instable especially under light irradiation and in the presence of oxygen. As for the monoradicals, also chlorination appears to be a favorable route to render the different diradicals stable. For the non-chlorinated and the perchlorinated (**PTH**) Thiele radicals, the ground states have been confirmed to be singlet states, with little to no diradical character (*y*_0_ = 0) [90–91], while more recent investigations have produced *y*_0_ = 0.3 for the non-chlorinated Thiele diradical [92]. Interestingly, the Thiele hydrocarbon without chlorination in the *meta*-positions of the four peripheral phenyl-rings (**TTH**), exhibits higher *y*_0_ of up to 0.4, indicating the mesomeric equilibrium to be further on the open-shell side (see Figure 12a) [92]. Also the perfluorinated version of the Thiele hydrocarbon (**TFC**) has comparable diradical character of *y*_0_ = 0.35 [93]. This increased diradical character has been attributed to increased structural flexibility in **TTH** and **TFC**. Their C–C bonds are longer than in the non-chlorinated Thiele radical, and **TTH**, and **TFC** are not as sterically congested as **PTH**. In **TTH**, the photoluminescence shifts with increasing solvent polarity while the ϕ increases from 69% in cyclohexane (λ_em_ = 691 nm) to 83% in chloroform (CHCl_3_) and 84% in toluene (λ_em_ = 716 nm) (see Figure 14a). This shift in the emission wavelength points towards a CT excited state. Apparently upon excitation, symmetry breaking charge separation [94] occurs leading to a (zwitterionic) CT excited state with a diarylmethylene unit acting as a donor and a trityl unit acting as an acceptor [92]. This symmetry breaking charge separation seems to be supported in solvents of higher polarity. By contrast, when the polarity of the solvent is increased beyond that of toluene, the ϕ drops to about 10% in THF (λ_em_ = 785 nm), which is typical for CT excited states that are stabilized by polar solvents (see Figure 14a) [92].

Similar observations can be made for the Chichibabin congeners **PTM-PTM** and **TTM**-**TTM** (see Figure 12a). The perchlorinated **PTM-PTM** exhibits a strongly twisted diphenyl bridge and therefore almost no conjugation between the two radical centers (*y*_0_ = 0.998) (see Table 3) [90,95]. The negative Δ*E*_ST_ indicates a singlet ground state; however, singlet states should not produce EPR signals (see Figure 12b). The yet obtained EPR signal indicates that the triplet state is thermally accessible, which is reasonable considering the small Δ*E*_ST_ of <0.1 kcal mol^−1^ (as determined by us on the UB3LYP/def2-SVP level of theory as part of this review, see Table 3). Cooling leads to a reduction of the intensity of the central EPR signal, fine structure, and significant diradical anisotropy, indicating that the triplet state becomes less and less populated (see Figure 12b) [95].

**Table 3:** Electronic and magnetic properties of selected diradicals. Values determined in cyclohexane (CH) unless stated otherwise.

in CH	**TPA(Me) (PyBTM)** **_2_**	**DR1**	**TTH**	**TTM-PhTTM**	**(TTM-Cz)_2_-An** (in toluene)	**TTM-TTM** (in toluene)	**PTM-PTM** (in CHCl_3_)	***m*** **-PTH** ^a^	**(Mes** **_2_** **-TTM)** **_2_** **-** ***m*** **Fl**

λ_abs_ [nm]	≈385	375	498	375	375	370	386	387	620
λ_em_ [nm]	667	654	691	671	≈750	780	–	610	635
ϕ [%]	7.9	16	69	0.4	3	0.8	–	1.4	92
*y*_0_	−	0.947	0.33–0.4	0.9	0.9	0.31–0.35 (crystal structure) 0.58 (unconstrained)	0.998^a^	0.78^a^	–
Δ*E*_ST_ [kcal/mol]	−11.9	−0.022	–	−1.51	–	(−6.62) – (−1.22) depending on technique	<0.1^a^	1.6^a^	−0.001

^a^Values determined by us experimentally or theoretically using UB3LYP/def2-SVP.

By contrast, the **TTM**-**TTM** analogue without chlorine substitution in the *meta*-positions of the phenyl rings has a perfectly flat biphenyl bridge and therefore a much-reduced diradical index (*y*_0_ = 0.58) [97]. The increased conjugation of the flat diphenyl bridge in **TTM**-**TTM** is responsible for the reduced diradical character and leads to a shift of the emission into the near-IR (λ_em_ = 780 nm, ϕ = 0.8% in toluene). While the ϕ of **TTM**-**TTM** is low, no photoluminescence has been reported for **PTM**-**PTM**. Δ*E*_ST_ of **TTM**-**TTM** is at −1.22 kcal mol^−1^ indicating a singlet ground state as well; however, the value is larger than for **PTM**-**PTM**, requiring heating to observe increased population of the triplet state and strengthening of respective EPR signals [97].

Further extension of the hexadeca-chlorinated diradicals (**TTH**, **TTM**-**TTM**) yields Müller’s **TTM-PhTTM** diradical (see Figure 12a) [96]. The additional phenylene ring disturbs the conjugation between the radical centers sufficiently to increase the diradical character to *y*_0_ = 0.9. DFT corroborates that the three bridge phenylenes are twisted against each other by about 35°. Δ*E*_ST_ has been determined from EPR measurements to be −1.51 kcal mol^−1^, whereas DFT gave −0.11 kcal mol^−1^. This Δ*E*_ST_ interval indicates that the triplet state is thermally accessible, represented by the improving EPR signal with increasing temperature (see Figure 12c) [96].

Increasing the distance between the radical centers even further by extending the bridge as in the case of **bPTM**, yields a diradical with full diradical character *y*_0_ ≈ 1 and minute Δ*E*_ST_ of order 10^−7^ kcal mol^−1^ (see Figure 12a) [98]. No photoluminescence has been reported for **bPTM**; however, this diradical shows extremely long coherence times of order *T*_1_ = 1 s (thermalization, spin lattice relaxation) and *T*_M_ ≈ 67 µs (phase memory time), both determined in carbon disulfide as a solvent and at low temperatures of 7 K and 15 K, respectively. These staggeringly long coherence times showcase the potential of precisely designed diradicals for molecular quantum applications [98].

#### Non-Kekulé diradicals with broken conjugation

The non-Kekulé equivalent to the Thiele diradical is represented by the Schlenk–Brauns diradical. The more stable perchlorinated version of this molecule (***m*-PTH**) has been reported in a set of publications [99–101]; however, while this body of work reports the most extensive characterization of these molecules, it has hardly been picked up thereafter (see Figure 13). A “***m*-TTH**” version of the diradical without chlorine substitution in the *meta*-positions, at least in the peripheral rings has not been reported to date. ***m*-PTH** exhibits a triplet ground state, represented by a positive Δ*E*_ST_ = 1.6 kcal mol^−1^ and a diradical character of *y*_0_ = 0.78 (as determined by us on the UB3LYP/def2-SVP level of theory as part of this review). While no photoluminescence data has been reported for ***m*-PTH** in the original publications, we here report a ϕ of 1.4% (in cyclohexane).

The *meta*-coupled equivalent to the Chichibabin radical has only been explored theoretically. Here, the radical centers are also connected by a biphenyl linker; however, not through the *para*-positions like in the Chichibabin diradical but through the respective *meta*-positions of the phenyl rings (with regard to the phenyl–phenyl connection) (cf. **TTM**-**TTM** in Figure 12) [102]. The resulting non-Kekulé diradical belongs to the group of alternant hydrocarbons and is supposed to exhibit a triplet ground state like *m*-**PTH** but with relatively high ϕ, offering the opportunity for optically detectable magnetic resonance (ODMR).

The next heavier homologue – the *meta*-coupled isomer of the Müller diradical (**TTM-PhTTM**) – has been synthesized as **TTM-*m*Ph-TTM** (see Figure 13). Δ*E*_ST_ for this **TTM-*m*Ph-TTM** diradical has been determined by DFT to be positive, indicating a triplet ground state; however, the value is smaller than the error of the calculation method, so it is fair to say that effectively, the energies of the singlet and triplet ground states can be considered degenerate. **TTM-*m*Ph-TTM** has a ϕ of 0.6% and the authors have shown that ODMR can be performed on an ensemble of molecules [103]. The next electronic homologue has been synthesized as a 3,6-fluorene-bridged diradical ((**Mes_2_-TTM**)**_2_-*m*Fl**, see Figure 13). This molecule is capped with electron-donating mesityl groups and exhibits a high ϕ of 92% in toluene solution. However, the molecule exhibits a singlet ground state. Again, Δ*E*_ST_ is so small that the singlet and triplet states have to be considered effectively degenerate (see Figure 13) [104]. Nevertheless, ODMR could be performed on the ensemble of diradicals, with a thermally accessible triplet state. The concept of *meta*-coupling of trityl radicals to produce non-Kekulé diradicals has also been followed by coupling two trityl radicals to the 1 and 6 positions of a perylene bisimide (**1,6-TTM-PBI**, see Figure 13), as well as through the 3 and 6 positions of *N*-phenylcarbazole (carbazole can be considered non-Kekulé, see explanation below) [105–106]. Despite the *meta*-coupling, these diradicals also exhibit negative Δ*E*_ST,_ meaning that the ground state is a singlet. From this series of diradicals with extending bridge length the spin state of the ground state can be rationalized, when considering that the dipolar coupling strength between the two radical electrons decays quickly. While the distance between the methine groups, at which the radical electrons reside in ***m*-PTH** are approximately 0.5 nm apart, in 1,6-**TTM-PBI** the distance between the radical sites is about 1.4 nm (also 1.4 nm in the (**Mes_2_-TTM)_2_-*m*Fl**). Apparently, the interaction between the electrons is too weak at these distances to allow them to assume a triplet state. However, the *meta*-coupling effectively hinders conjugation and so **1,6-TTM-PBI** has a strong diradical character of *y*_0_ = 0.966 (see Figure 13).

While triarylamines are typically sp^2^-hybridized and the electrons on the nitrogen are delocalized to a substantial degree, in triphenylamine- or carbazole-bridged diradicals it is impossible to draw a correct Kekulé structure (see Figure 13). Therefore, such triphenylamine- or carbazole-bridged diradicals, can be considered non-Kekulé hydrocarbons. Triphenylamine-bridged **PyBTM** diradicals have been synthesized and the *para*-substitution in the free phenylamine ring has been functionalized with varying electron-accepting or donating units (see Figure 13). It has been shown that the absolute Δ*E*_ST_ value increases when going from electron-withdrawing units – which reduce the UB3LYP calculated spin-density at the trityl sites and increase the exchange coupling – to electron-donating units (see Figure 13). Altogether, Δ*E*_ST_ is negative, corroborating a singlet ground state at the large distance (≈1.6 nm) between the radical centers [107]. Since triphenylamine has an overall electron-donating effect, it is not surprising that all **TPA(PyBTM)_2_** diradicals exhibit acceptable ϕ of up to 7.9% for the methyl-substituted **TPA(Me)(PyBTM)_2_**_,_ where the triphenylamine has the strongest electron donor character [107]. The variation of the electron directing unit in the *para*-position of the free phenylamine ring also allows shifting of the emission color from λ_em_ ≈ 700 nm for **TPA(CN)(PyBTM)_2_** to λ_em_ ≈ 790 nm for **TPA(OMe)(PyBTM)_2_** (see Figure 14b)_._ This bathochromic shift for electron-donating units is analogous to the well-studied effect of increasing electron donation in monoradicals [63,65].

Interestingly, when two **TTM** radicals are coupled via a carbazole unit, Δ*E*_ST_ shrinks to −0.022 kcal mol^−1^, while *y*_0_ ≈ 1 as expected for non-Kekulé diradicals (see **DR1** in Figure 13) [108]. **DR1** shows ϕ of 16% and relatively short luminescence decay of *τ* = 10.6 ns compared to **TTM-Cz** with τ ≈ 40 ns (ϕ = 88–91%, see Figure 8). Knowing that the luminescence decay scales with the ϕ in donor-functionalized trityl radicals, the performance of the carbazole-bridged diradical can be rationalized. The photoluminescence spectrum of **DR1** (λ_em_ = 654 nm) is red-shifted against that of **TTM-Cz** (λ_em_ = 628 nm) and its shape resembles more the spectrum of **TTM-3PCz** (λ_em_ = 664 nm), which is also a structural sub-motif of **DR1** (cf. Figures 6, 13 and 14c) [108]. **DR1** shows magnetoluminescence, meaning that the films of 0.5 wt % of **DR1** in **PMMA** show increasing photoluminescence, when increasing the magnetic field from 0 to 7 T at 2 K. The authors explain the magnetoluminescence by improved radiative relaxation from excited triplet states than singlet states and the spin-forbidden ISC between these excited states. In the magnetic field, the spins align and the triplet state population is increased (similar to temperature dependent photoluminescence). Population of the lowest triplet state allows reaching of excited triplet states that exhibit better radiative relaxation rates than the corresponding singlet excitons.

Bridging of two **TTM-Cz** with an anthracene unit does not deliver a photoluminescence spectrum that can be explained by a single substructure of this molecule. Instead, the emission of (**TTM-Cz)_2_-An** is much further red-shifted than **TTM-Cz** or any other sub-fragment (see Figure 13 and Figure 14d) [109]. The ϕ of 3% is surprisingly low, considering that the other diradicals with donor–acceptor subunits have produced much higher values. Apparently, the first electronically excited state is not the expected CT state between the carbazole fragment and the **TTM** unit, but instead an excited triplet state on the anthracene unit. Since the distance between the radical sites is ≈2.2 nm it is not surprising that the ground state of this molecule represents a singlet state, as there is little to no dipolar coupling between the radical electrons. However, once excited the molecule exists in a quintet state, which explains the broad and redshifted emission spectrum. The excited anthracene in its triplet state leads to spin-polarization of the neighboring **TTM-Cz** units to an overall (four-spins) quintet state (the **TTM** radical site to anthracene distance is ≈1 nm). Interestingly, the two radical electrons on the **TTM** sites remain correlated for about 30 µs even after the molecule has electronically relaxed and the anthracene has returned to its singlet ground state. This ground state polarization is possible at room temperature with the (**TTM-Cz)_2_-An** molecules dispersed in a **PMMA** matrix [109]. These properties render the molecule capable for application as a spin-optical interface for optical initialization of qubits and potentially optical readout of the spin-state.

Bridges between trityl radicals can also be employed to align the radical centers in a cage geometry so that the trityl units are oriented on top of one another, with the radical p-orbitals pointing towards each other (see **PTM-C** in Figure 13) [110]. The crystal structure of this molecule allows determination of the distance (0.99 nm) between the two (bridgehead) carbons, where the radical electrons reside. Because there is no conjugation possible through the biscarbazole bridges and because the distance between the radical sites is small, the ground state of this molecule is a triplet as indicated by the positive Δ*E*_ST_ = 0.005 kcal mol^−1^ determined experimentally. By contrast, unrestricted DFT delivered a singlet ground state with a similarly small energy gap, substantiating that the triplet and singlet states can be considered degenerate. Despite the threefold carbazole substitution, there is no photoluminescence reported for this diradical.

#### Summary: diradicals based on the triarylmethyl motif

In diradical systems, the conjugation between the radical centers plays an important role. In the family of Kekulé diradicals we see a clear correlation between the number of phenyl rings between the radical centers and the diradical index *y*_0_ (see Table 3). The diradical character is small for **TTH** (*y*_0_ ≈ 0.4) and steadily grows for **TTM**-**TTM** (*y*_0_ = 0.58), **TTM-PhTTM** (*y*_0_ = 0.9) to **bPTM** (*y*_0_ ≈ 1). By contrast, non-Kekulé diradicals, which do not allow conjugation have in principle higher diradical character.

Diradicals may present the smallest viable building blocks for organic quantum technology. **bPTM** exhibits coherence times that can rival or even outperform state-of-the-art solid-state inorganic systems for quantum computing [98]. By contrast, non-Kelulé diradicals bear the potential of exhibiting a triplet ground state, and therefore an electronic structure that resembles that of nitrogen vacancies (NV) in diamond. Such molecular NV-centers represent an interesting platform for quantum sensing, where for example small magnetic fields can be detected. This magnetic field sensing is important for applications ranging from future navigation to medical imaging systems.

Unfortunately, only few of the diradicals present today exhibit photoluminescence and those that do have no distinct triplet ground state. Photoluminescence is an important means for reading out the spin state in such molecular quantum systems, which is why this property should be reflected in future research endeavors in this field. The cases of **TTH**, with the highest ϕ among the Kekulé radicals and **(Mes_2_-TTM)_2_-*m*Fl** among the non-Kekulé radicals show that it requires CT excited states for efficient emission. The strong emission of open-shell molecules that exhibit CT states is known from the trityl monoradicals; however, to date only few attempts have been reported, where CT excited states have been designed into trityl-derived diradicals.

### Triarylmethyl multiradicals

For molecules with more than two unpaired electrons, the singlet and triplet ground states nomenclature is no longer correct. For such multiradicals the concepts of ferro-, antiferro-, and paramagnetism are more accurate descriptors [111]. On the one hand, in *ferromagnetic* molecules neighboring magnetic moments align parallel to each other, resulting in a strong overall magnetic alignment. In typical ferromagnets this alignment persists even in the absence of an external magnetic field (this state would correspond to a diradical with triplet ground state). On the other hand, *antiferromagnetic* molecules exhibit neighboring magnetic moments that align antiparallel to each other. This leads to a cancellation of the overall magnetic moment, resulting in minimal macroscopic magnetization (corresponding to what we discussed for diradicals with a singlet ground state). By contrast, in *paramagnetic* materials, no alignment without the presence of an external magnetic field can be observed.

Often, ferromagnetic and antiferromagnetic molecules exhibit strong magnetic interactions between adjacent electron spins only at low temperatures but lack a net magnetic moment at room temperature where they behave as paramagnets.

**Figure 15:**
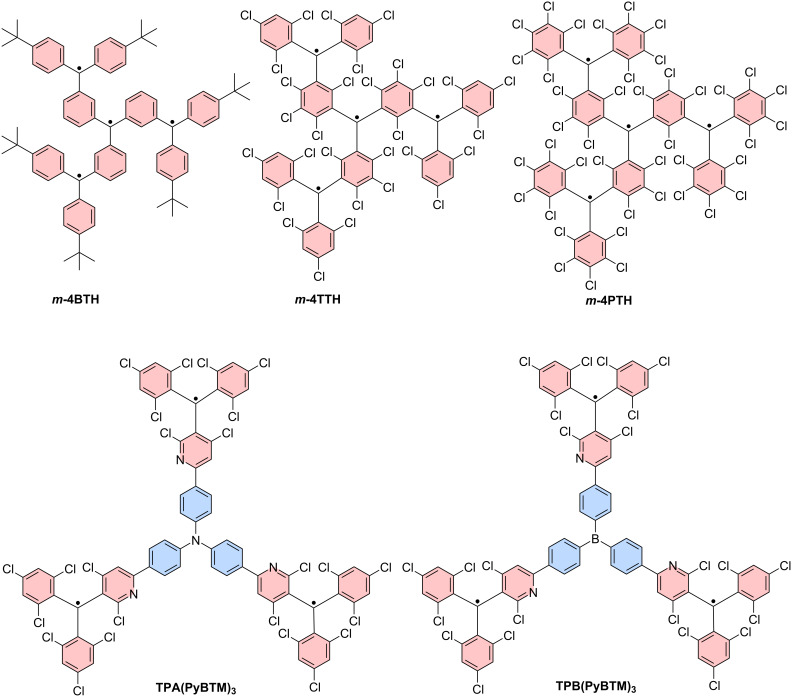
Molecular structures of ***m*-4BTH** (*meta*-butylated Thiele hydrocarbon), ***m*-4TTH** (*meta*-trichlorinated Thiele hydrocarbon) and ***m*-4PTH** (*meta*-perchlorinated Thiele hydrocarbon) tetraradicals and triradicals **TPA(PyBTM)_3_** and **TPB(PyBTM)_3_**.

In the past, much effort has been steered into the synthesis of trityl-derived multiradicals. *Meta*-linked multiradicals are of special interest as their broken conjugation should help facilitate high-spin ground states and therefore ferromagnetic coupling. In *meta*-linked diradicals this has already been observed in the form of triplet ground states in the aforementioned ***m*-PTH** [112–114]. By contrast, *meta*-linked multiradicals have proven to be rather elusive, due to their impressive steric congestion [115]. Despite these synthetic challenges, three *meta*-linked tetraradicals ***m*-4BTH**, ***m*-4TTH** and ***m*-4PTH** have been reported, which exhibit the proposed high spin (quintet) ground state (see Figure 15) [100,111,115–116]. Moreover, a similar *para*-linked ***p*-4BTM** terminated with galvinoxyl groups has been realized [117]. No photoluminescence has been reported for these tetraradicals.

The above described triphenylamine bridged diradicals, have also been reported in the form of triradicals, either with triphenylamine or with triphenylborane as non-Kekulé coupling nodes for the radical moieties [107]. While **TPB(PyBTM)_3_** exhibits a ϕ of only 0.3%, **TPA(PyBTM)_3_** features a much better ϕ of 6.1%. This is due to the electron-donating character of the triphenylamine moiety, as opposed to the electron-poor triphenylborane node. The electron-accepting character of triphenylborane in the triradicals therefore induces a photoluminescence maximum at shorter wavelengths **TPA(PyBTM)_3_** (<700 nm), as compared to the emission maximum of the triradical with the electron-donating triphenylamine node (>700 nm) (cf. purple and dark green photoluminescence spectra in Figure 13b). While for both triradicals the high and low spin states are almost degenerate in energy, the multiplicity has almost no effect on the photoluminescence, rendering such weakly coupled multiradicals useful for optoelectronic applications [107].

#### Polymer chains

Polymerization of radical-containing monomers represents a complementary approach to produce multiradicals without running into problems of steric congestion.

**Figure 16:**
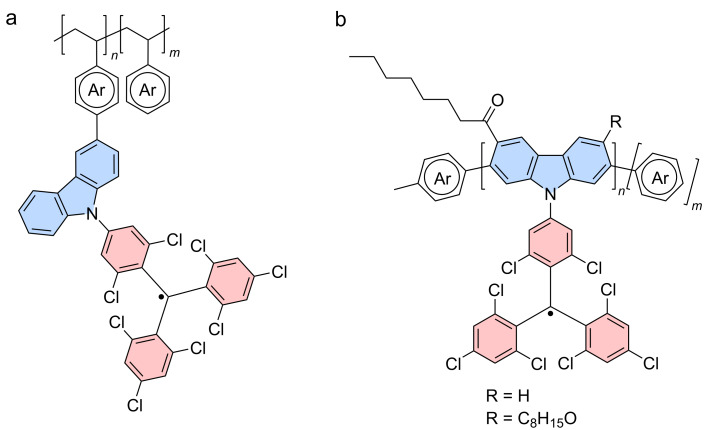
a) Polystyrene-based **TTM-Cz** polymer. b) Molecular structure of radical particles with backbone through the **Cz**-derived donor of **TTM-Cz**.

Interestingly, co-polymerization with non-radical molecules allows for dilution of the spin species or dedicated charge- or energy transfer phenomena between these units and the respective trityl radical. This way, a styrenyl **TTM-Cz** monomer has been incorporated into a polystyrene backbone and employed as the light-emitting layer in an OLED (see Figure 16a) [118–119]. Moreover, **PTM** has been employed to terminate a polyphosphorhydrazone dendrimer, which can be reversibly switched electrochemically between a multiradical state and a multi-anionic state with optical readout [120]. In a different approach, 2,7-dibromocarbazole with *N*-coupled **TTM** has been subjected to a C–C cross-coupling reaction to obtain conjugated polymer nano- and microparticles (see Figure 16b) [121]. The particles exhibit a ϕ of up to 28.1% and those with 50 mol % of radical are paramagnetic even at low temperatures, documenting the amorphous morphology and the resulting magnetic anisotropy of the radical species inside the particles.

**Figure 17:**
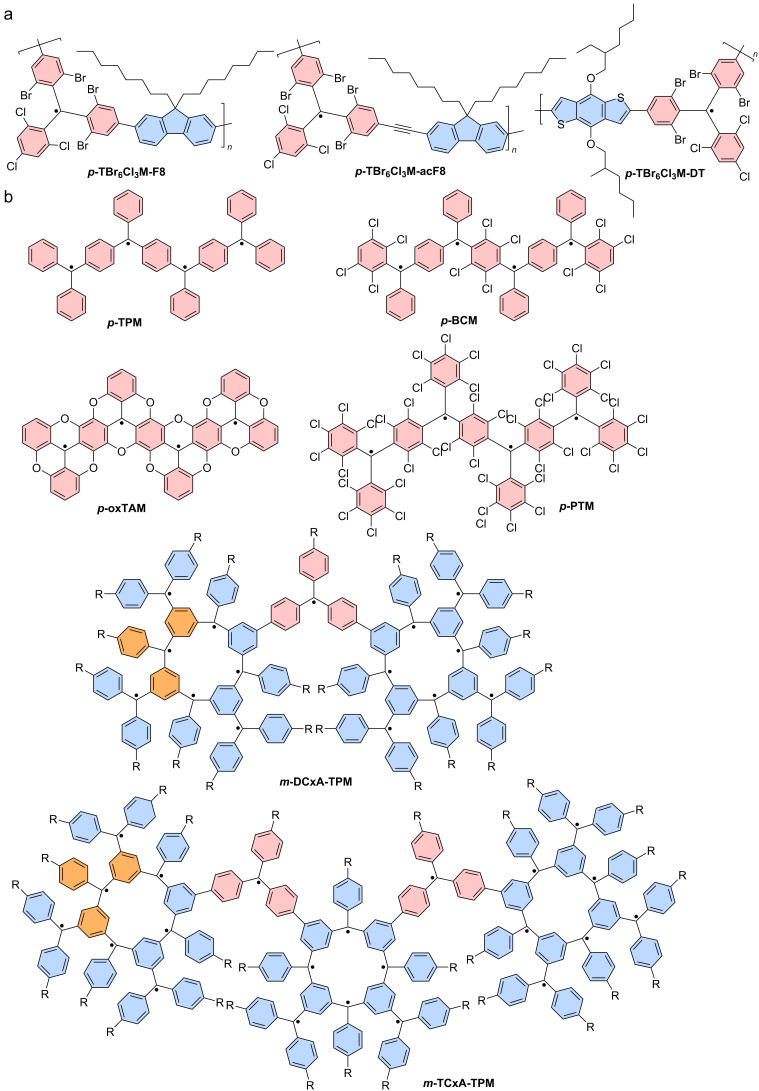
Molecular structures of polyradicals. a) Molecular structures of ***p*-TBr_6_Cl_3_M-F8**, ***p*-TBr_6_Cl_3_M-acF8** and ***p*-TBr_6_Cl_3_M-DT**. b) Molecular structures during the theoretical investigation of ***p*-TPM** (top left), ***p*-BCM** (top right), ***p*-oxTAM** (bottom left) and ***p*-PTM** (bottom right). c) Calix[4]arene multiradicals ***m*-DCxA-TPM** (top) and ***m*-TCxA-TPM** (bottom). (*met*a-connected triphenylmethyl subunit in calixarene highlighted in orange, *para*-connected bridging trityl unit highlighted in red).

Incorporation of **TTM** into the backbone of polymeric materials can be achieved by co-polymerization using fluorene and dithiophene compounds by Sonogashira, Stille, and Suzuki coupling (see Figure 17a). Incorporation of trityl radicals into the conjugated backbone can lead to conjugation effects, in which some of the radicals may become consumed in closed-shell quinoidal structures [50,122]. This effect becomes apparent in the luminescence spectra of the particles that are much broader than expected from the monomeric units. The resulting polymers exhibit weak emission between 600–1100 nm [50,122].

This problem of radical combination through quinoid formation in conjugated radical polymers has been investigated in a theoretical approach. Using DFT calculations, conjugated trityl polymers have been investigated with regard to their geometry, dihedral angles, and ground state energies for both the open- and closed-shell variations (see Figure 17b). Interestingly, the authors of the study include different structural changes to tune the dihedral angle ‹*ω*› between two radical methyl planes. The ether-annulated ***p*-oxTAM** is completely flat (‹ω› = 0°), whereas the perchlorinated ***p*-PTM** polymer has the largest dihedral angle (‹ω› = 45.8°) due to steric demand of the large chlorine substituents (see Figure 17b). Naturally, ***p*-TPM** exhibits the greatest degree of freedom and the minimal energy ground state geometry exhibits a dihedral angle (‹ω› = 26.5°) between those of ***p*-oxTAM** and ***p*-PTM** [123]. In analogy to the diradical character *y*_0_, in polyradicals we can express the radical–quinoidal balance as the average spin population ‹|µ_αC_|›. ‹|µ_αC_|› increases with ‹ω›, indicating that planarity and conjugation favor the quinoidal structure and radical combination. Non-symmetric substitution like in ***p*-BCM** leads to variable dihedral angles, seemingly favoring the quinoidal structure as well.

Dendritic calixarenes ***m*-DCxA-TPM** and ***m*-TCxA-TPM** composed of *para*- and *meta*-connected trityls exhibit ferromagnetic domains within the molecules, which are termed spin clusters with large *S* (see Figure 17c). The calixarene radical clusters (blue in Figure 17b) possess total spin *S* = 7/2 or 6/2 and are bridged by triarylmethyl monoradical linkers (red in Figure 17b) with *S* = 1/2. The spin clusters are randomly coupled ferro- and antiferromagnetically through the triarylmethyl linkers [124]. ***m*-DCxA-TPM** and ***m*-TCxA-TPM** can be viewed as di- and trimeric subunits of a longer spin-chain, and could therefore present an interesting future endeavor for potential conjugated polymers with strong magnetic ordering.

#### Supramolecular frameworks

While oligomers and polymers often assume a disordered coiled geometry or an amorphous morphology in the solid state, trityl-based radicals can also be assembled into highly ordered molecular frameworks. Such supramolecular frameworks are a class of material, formed under reversible association conditions using non-covalent interactions, like hydrogen bonds, π–π interactions, metal-coordination, or electrostatic forces. The resulting molecular scaffolds can vary in their dimensionality (two- or three-dimensional), in size and their homogeneity, and in their properties. Molecular frameworks exhibit a porous structure, rendering these materials interesting for gas adsorption and catalysis. Moreover, spin interaction between the unpaired electrons of radical-containing molecular frameworks can be useful for their application in spintronics or quantum information processing. In the following, we will discuss hydrogen-bonded radical supramolecular organic frameworks (SOFs) and metal coordinated organic frameworks (MOFs).

**Figure 18:**
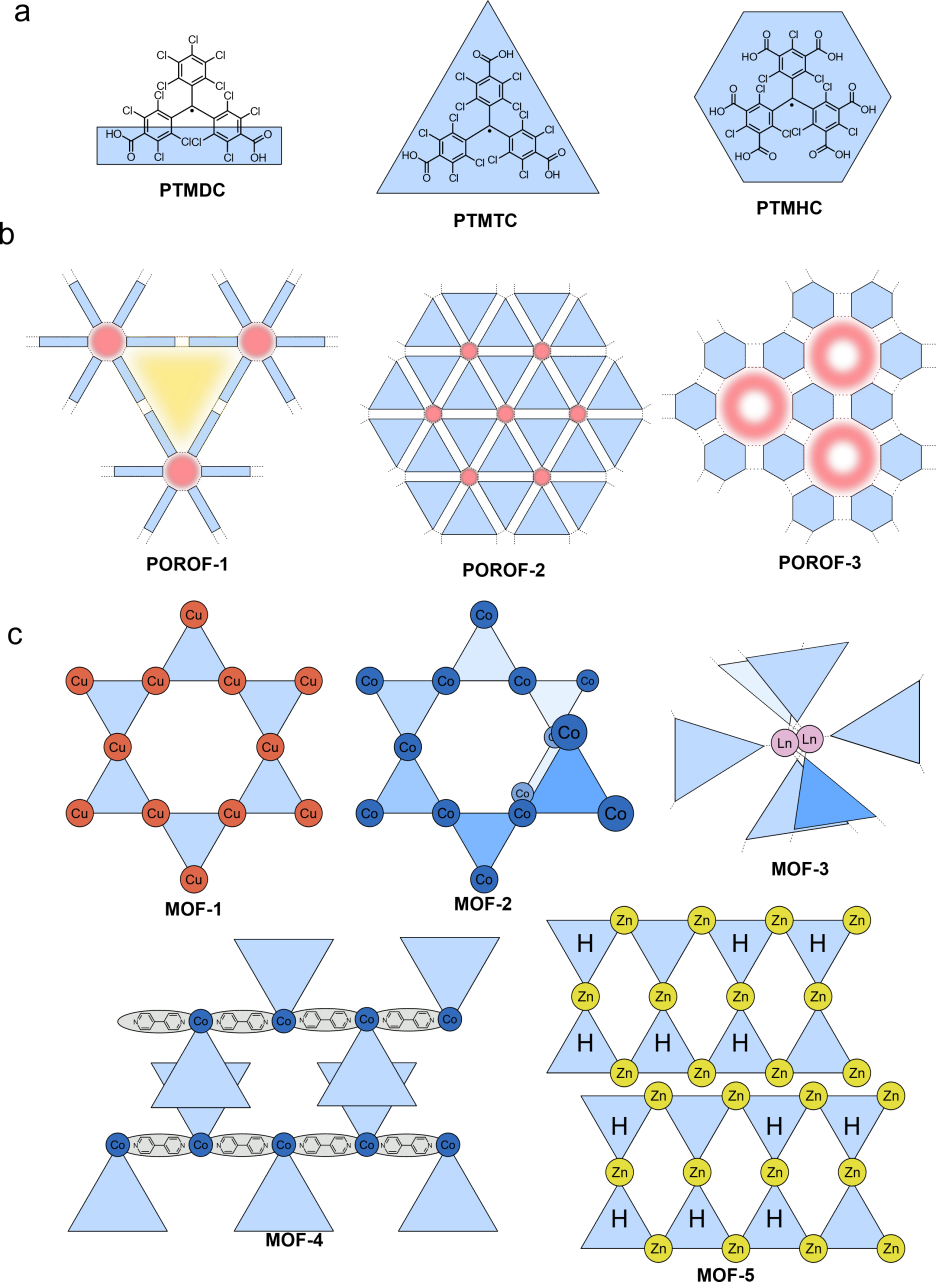
Structures of coordination and metal-organic frameworks. a) Carboxylic acid functionalized monomers. **PTMDC** (left), **PTMTC** (middle), and **PTMHC** (right). b) Coordination frameworks based on carboxylic acid functionalized monomers, connected by hydrogen bonding (dashed lines) **POROF-1–3**. Red and yellow markings represent hydrophilic and hydrophobic channels, respectively. c) Simplified structures of MOFs based on **PTMTC** with copper, cobalt, zinc and Ln = Tb, Gd, Eu. **MOF1–5** (hydrogenated, closed-shell **PTMTC-H** ligands are indicated by a triangle with a central H) (water and other small ligands omitted for clarity).

**Hydrogen-bonded frameworks:** Trityl-derived radicals with carboxylic acid moieties in *para*- or *meta*-positions of the phenyl groups have been employed regularly to design and produce different types of hydrogen bonded trityl SOFs (see **PTMDC**, **PTMTC** and **PTMHC** in Figure 18a and resulting network structures in Figure 18b).

The implementation of these open-shell building blocks with supramolecular (carboxylic acid) recognition motifs shows several benefits. The bulky structure of highly chlorinated TPM radicals prevents a close packing between units and enables exchange interaction via the hydrogen bond without leading to radical combination and quinoid formation, as is often the case for *para*-coupled trityl radicals. **POROF-1**, which has been crystallized from (twofold carboxylated) **PTMDC**, forms 2-dimensional hexagonal structures with smaller *hydrophilic* pores, due to the connecting carboxylic acid groups. The local hexagonal structures organize into regular patterns with long range regularity, forming also larger *hydrophobic* pores (see Figure 18b, yellow shading). These 2-dimensional layers stack on top of each other, forming channels, large enough to host for example *n*-hexane solvent molecules [125]. The (threefold carboxylated) **PTMTC** also forms layered hexagonal structures of similar pore size [126]. However, here the larger pores are hydrophilic, preventing the ingress of non-polar solvent guest molecules into the channels of this **POROF-2** framework (see Figure 18b, red shading).

Magnetic susceptibility (χ) measurements show mostly paramagnetic behavior of both **POROF-1** [125] and **POROF-2** [126], with effectively identical χ*T* values of 0.38 emu K mol^−1^ at 300 K (χ*T* for uncorrelated spins (S = 1/2) is expected at 0.375 emu K mol^−1^). At temperatures below 50 K weak antiferromagnetic exchange interactions can be observed for **POROF-1**. The magnetic properties are impervious to the presence of solvent guest molecules. By contrast, **POROF-2** reveals slightly increased χ*T*-values at 5 K and a ferromagnetic ordering of the unpaired radical electrons at 0.11 K [126].

The crystalline structure of hexa-*meta*-carboxlyated **PTMHC** framework **POROF-3** has been observed to incorporate solvent molecules during the crystallization process via hydrogen bonding (see Figure 18b). Different framework morphologies can be obtained depending on the solvent system, from which the frameworks are crystallized [127]. In the case of **THF**, every carboxylic acid group is bound to one **THF** molecule and the resulting **[PTMHC∙(THF)_6_]** clusters self-assemble into a honeycomb lattice.

For **Et_2_O** as a solvent, only half of the carboxylic acid groups are bound to the solvent, rendering three of these units (one per phenyl ring) accessible for hydrogen bonding between neighboring **[PTMHC∙(Et_2_O)_3_]** clusters. Due to the lack of direct hydrogen bonding between the **PTMTC** units, **[PTMHC∙(THF)_6_]** exhibits purely paramagnetic behavior between 10–200 K and very weak antiferromagnetic behavior below 10 K, whereas **[PTMHC∙(Et_2_O)_3_]** shows weak ferromagnetic interactions at low temperatures.

Unfortunately, photoluminescence has not been reported for any of the mentioned hydrogen-bonded frameworks, although one could assume that the emission behavior would be similar to the photoluminescence of the respective building blocks, which has been reported to be around 6% (in CHCl_3_) [128]. The rigidity of the produced SOF material could lead to enhanced ϕ, considering that radicals are immobilized in a crystalline environment of their hydrogenated closed-shell parent molecules, which reduces non-radiative (vibrational) relaxation pathways [43].

**Metal-coordinated radical frameworks:** The above discussed carboxylic acid functionalized **PTM** derivatives have not only been used in hydrogen bonded SOFs, but also for the generation of MOFs [128–133]. Copper (Cu) and cobalt (Co) ions form honeycomb arrangements together with **PTMTC**, with the metal center residing on the hexagonal grid (see **MOF-1** in Figure 18c). While the planar coordination to Cu ions results in 2-dimensional layers of **MOF-1**, the coordination to Co ions yields a helical tertiary structure in **MOF-2** (see Figure 18c) [130–131]. When a second 4,4’-bipyridine ligand is admixed, the Co ions restrict the **PTMTC** radical building blocks into quasi-1-dimensional strands, where the Co ions are coordinated octahedrally (see **MOF-3** in Figure 18). Each metal is additionally bound to one **PTMTC** radical ligand and three water molecules (omitted in Figure 18 for clarity). The non-coordinated carboxylic acid groups bind to water molecules of neighboring strands, bridging and aligning the chains into a quasi-parallel, stacked fashion [132].

Like in hydrogen bonded frameworks, there is virtually no information about the luminescence of trityl radical containing MOFs. **PTMTC** has been reported to bind to lanthanides, such as europium (see **MOF-4** in Figure 18) [129]. However, when introducing a **TTM** radical derivative as a linker for Ln-MOFs, the broad absorption of **PTMTC** at 500–575 nm overlaps with the characteristic emission bands of lanthanides, which may be the reason for emission quenching in these systems. Only for **MOF-5** photoluminescence is reported. **MOF-5** exhibits photoluminescence that is similar to that of the **PTMTC** building block in CHCl_3_ solution [128]. The Zn ions force the **PTMTC** ligands into ribbons, which stack in an off-set fashion (see Figure 18c). **MOF-5** is paramagnetic irrespective of the temperature. The paramagnetic nature might be caused by the low concentration of actual radical units, which are dispersed in a matrix of the α-hydrogenated **PTMTC-H** and the diamagnetic Zn(II) ions.

In **MOFs-1**, **-2** and **-3** a transition between ferro- and antiferromagnetic effects is typically observed at low temperatures. For the **MOF-4** (with Ln = Eu, Gd, Tb) antiferromagnetic behavior at low temperatures is reported, with a minimum χ*T*-value at around 2 K each. For the **MOF-4** with Ln = Eu a smooth decrease between 300 and 10 K is ascribed to a depopulation to close-to-ground-state excited states, whereas the more sharp decrease below 10 K is believed to be caused by antiferromagnetic radical–radical interaction through the lanthanide ions, as the distances are assumed to be too long to sustain through-space coupling [129].

**Figure 19:**
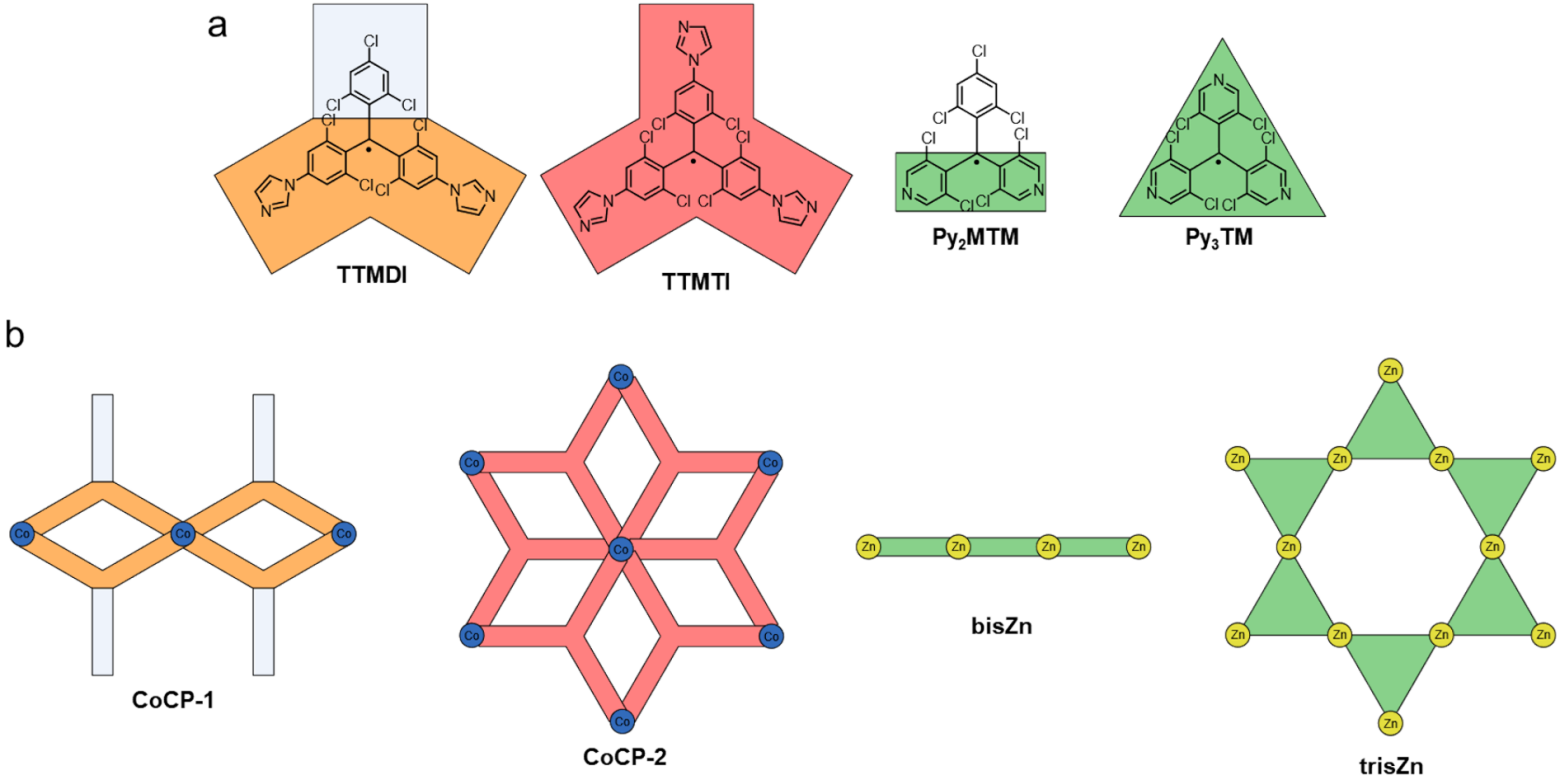
Structures of coordination and metal-organic frameworks. a) Molecular structures of monomers **TTMDI**, **TTMTI**, **Py_2_MTM** and **Py_3_TM** (from left to right). b) metal-organic framework structures **CoCP-1, CoCP-2, bisZn** and **trisZn** with cobalt and zinc as coordination centers.

The variety in trityl-derived MOFs has been further increased by using coordinating groups other than carboxylic acid units. **TTM** radicals have been *para*-substituted by imidazole units (**TTMDI**, **TTMTI**), or alternatively, the phenyl groups have been replaced by pyridines, which exhibit superior photostability as discussed above (see Figure 19a, and cf. to **PyBTM** in Figure 3). Using Co ions, a linear coordination polymer **CoCP-1** and a two-dimensional **CoCP-2** can be achieved with **TTMDI** and **TTMTI**, respectively (see Figure 19b) [134]. While no photoluminescence is reported for these MOFs, UV–vis spectra show broad absorption bands around 385 nm and 600 nm, resulting from the typical (in-phase and out-of-phase combinations of) HDMO–SOMO and SOMO–LUMO transitions of the trityl-based radical ligands [134]. Moreover, bands at 470 and 508 nm and a shoulder at 720 nm can be attributed to the octahedrally coordinated, high-spin Co(II) ions and the band at 553 nm to a coexistence between absorption of the radical and heavy atom effects of Co(II) [134].

Magnetic susceptibility measurements reveal higher χ*T*-values than expected, due to significant orbital contribution of the octahedral Co(II) ions. Lower temperature generates a continuous decrease in χ*T* down to 1.66 emu K mol^−1^ at 3 K for **CoCP-1** and 1.55 emu K mol^−1^ at 4 K for **CoCP-2**, indicating an antiferromagnetic coupling between ions and ligands, which is supported by DFT calculations [134].

The **Py_2_MTM** and **Py_3_TM** ligands produce two intriguing MOFs coordinated by Zn ions (see **bisZn** and **trisZn** in Figure 19b). While **Py_2_MTM** yields a linear metal-coordination polymer, **Py_3_TM** delivers a hexagonal 2-dimensional MOF [133]. Although the unpaired electrons of the trityl radicals are in even closer proximity to the coordinated metal center and towards each other than in the imidazole based frameworks **CoCP-1** and **CoCP-2**, χ*T* magnetic susceptibility reveals antiferromagnetic behavior at low temperature [133].

Photoluminescence of **bisZn** and **trisZn** at 4.2 K is negligible with ϕ values of <0.001 and 0.02%, respectively. However interestingly, magnetoluminescence is observed when an external magnetic field is applied [56,135–136]. It is proposed that the rigidity of the MOF reduces radical–radical interactions at low temperatures so that modulation of the spin sublevel population can be achieved by a magnetic field and read out by fluorescence [133]. By contrast, greater spacings between radical units do not seem to result in a similar effect, as magnetoluminescence has not been reported for any of the other SOFs or MOFs.

In summary, one ferromagnetic and one magnetoluminescent MOF have been reported in the literature. A clear understanding of what causes ferromagnetism in MOFs has not been reached. Ground state population of the Kramer’s doublet of Co(II) ions at very low temperature with *S*_eff_ = 1/2 could help as an explanation for the increased correlation of **MOF-4** at 1.8 K [131]. But as a coexistence of ferro- and antiferromagnetic interactions is reported for this compound, it is still elusive what causes such behavior. So far it is only clear that for magnetoluminescence to occur, a spatial separation of radicals needs to take place. But it remains unclear, at which distance this response breaks down. More insight needs to be gained on these topics and a design strategy needs to be developed to help improve magnetic and fluorescent properties of radical MOFs in the future. Concepts from the discrete radical constructs could help to improve the performance, like incorporation of donor moieties in the radical synthons to achieve higher quantum yields and possibly a combination of ferromagnetic and emissive properties.

**Figure 20:**
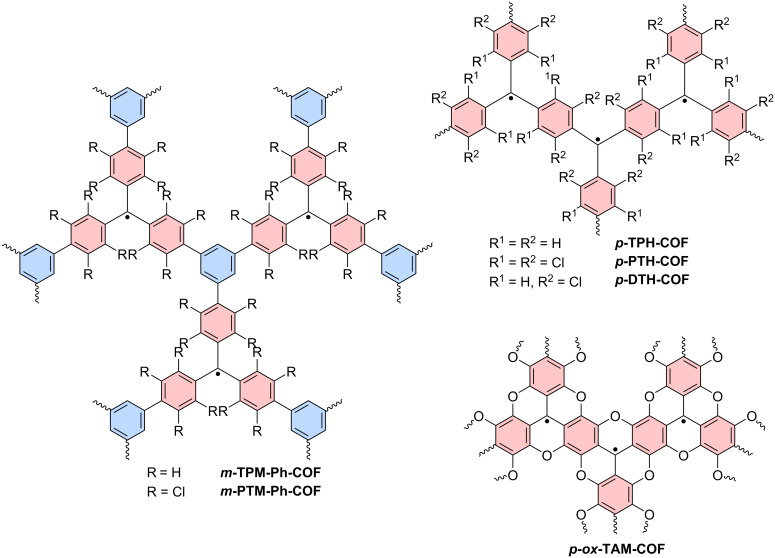
Molecular structures of covalent organic frameworks ***m*-TPM-Ph-COF**, ***m*-PTM-Ph-COF**, ***p*-TPH-COF**, ***p*-PTH-COF**, ***p*-DTH-COF** and ***p*-ox-TAM-COF**.

**Covalent organic radical frameworks:** Covalent organic frameworks (COFs) with covalent and non-reversible connections between trityl radical building blocks have been of particular interest, as they – much like SOFs and MOFs – combine tunable pore sizes and predictable geometry to address a wide set of potential applications. In contrast to SOFs and MOFs, the covalent connection comes with the well-known problem of the equilibrium between benzenoid multiradical and quinoidal closed-shell electronic structure in *para*-connected trityl building blocks. However, *para*-connection delivers the most predictable hexagonal COFs, which is why most reported COFs are based on *para*-connected building blocks. For this reason, there are several theoretical studies, which discuss whether the assembly delivers a significant fraction of unpaired (non-quinoid) radical electrons and what magnetic properties to expect from the material [137–140]. In theoretically explored COFs the change of the dihedral angles upon uniaxial stretching or out-of-plane compression is studied in chlorinated and non-chlorinated trityl units connected through 1,3,5-benzene (see ***m*-TPM-Ph-COF** and ***m*-PTM-Ph-COF** in Figure 20). It is observed that stretching of these COFs causes a flattening of the dihedral angles between neighboring phenyl rings, resulting in a higher orbital overlap. This orbital overlap gives rise to a shift from the open-shell to the closed-shell quinoidal structure. All ring-sharing, *para*-connected COFs, which represent an extension to the formerly discussed Thiele diradicals, have been explored theoretically and show an antiferromagnetic ground state in their open-shell configuration (see ***p*-TPH-COF**, ***p*-PTH-COF**, ***p*-DTH-COF** in Figure 20) [137,139–140]. For all these *para*-connected COFs, this open-shell ground state solution is lower in energy, except ***p*-TPH-COF** where both multi-radical and quinoidal solutions are nearly degenerated [137]. Interestingly, for ***p*-oxTAM-COF** with its fully planarized geometry (cf. ***p*-oxTAM** in Figure 17b), the localized quinoidal state spontaneously produces a delocalized semi-metallic state that could not be stabilized during the calculations [137]. However, when we introduce trifunctional crosslinkers so that the conjugation between trityl radicals becomes broken through the *meta*-connection (***m*-TPM-Ph-COF** and ***m*-PTM-Ph-COF** discussed before) ferromagnetic exchange interactions are found. However, the necessary spin alignment into high spin materials is expected only at temperatures below 10 K [138].

**Figure 21:**
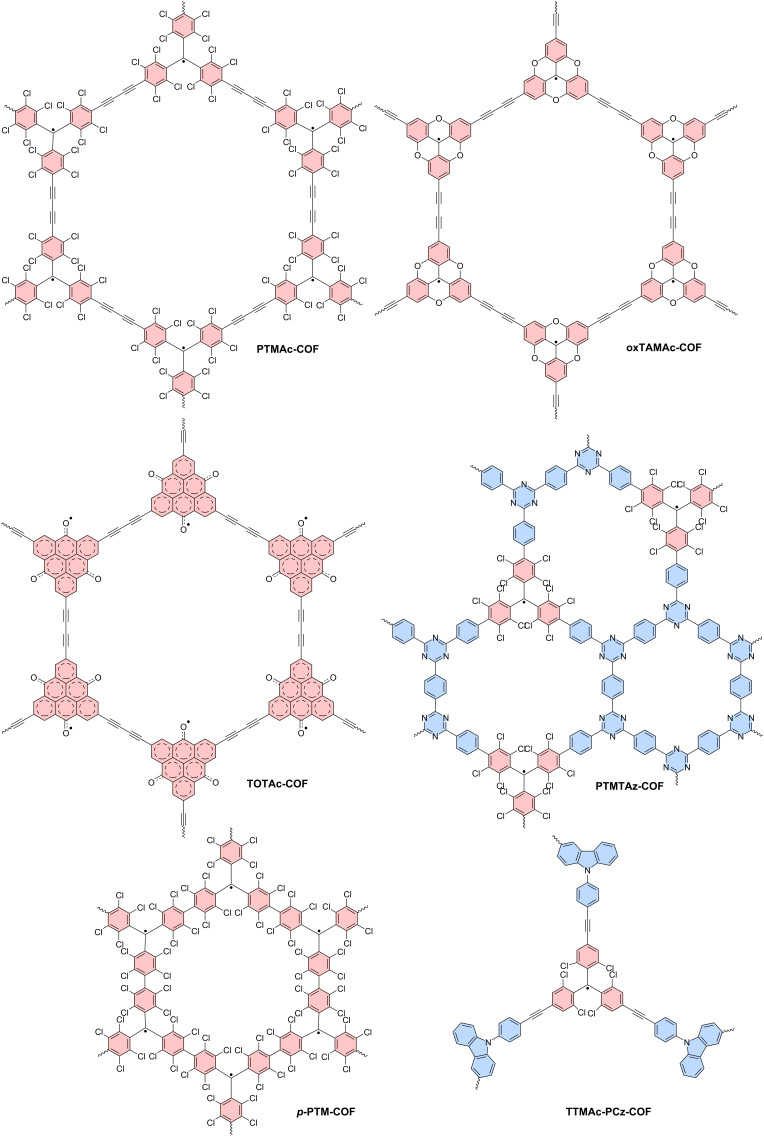
Molecular structures of covalent organic frameworks **PTMAc-COF, oxTAMAc-COF**, **TOTAc-COF, PTMTAz-COF**, ***p*-PTM-COF,** and **TTMAc-PCz-COF**.

**PTMAc-COF** has been synthesized in 2018 by two independent research groups [141–142]. Both groups observed Mott insulating properties, arising from the antiferromagnetic behavior below 42.5 K, which were also predicted by independent theoretical studies (see Figure 21) [140]. Decrease of spin localization from **PTMAc-COF** to **oxTAMAc-COF** and **TOTAc-COF** (due to their coplanar structures) has been observed to lead to higher exchange values and therefore more stable antiferromagnetic configuration compared to **PTMAc-COF** [140].

The above discussed experimentally investigated radical COF examples have been synthesized through C–C cross-coupling reactions [141–144]. Interestingly, when an additional coupling agent is included, the radical centers can be diluted in the “matrix” of electron-accepting crosslinker units, as is the case in **PTMTAz-COF** (see Figure 21) [145]. Paramagnetic behavior has been reported for this triazine-linked **PTMTAz-COF** [145]. Here the distance between neighboring spins, which is nearly 20 Å, is too large for significant spin–spin interactions to persist. Again, no emissive behavior is reported for these COFs. By contrast, the distance between unpaired electrons is significantly smaller in ***p*-PTM-COF** than in the triazine-linked **PTMTAz-COF**; however, ***p*-PTM-COF** shows paramagnetic behavior as well, arising from a coexistence of both ferro- and antiferromagnetic interactions at low temperatures [144]. Despite theoretical calculations predicting an open-shell conformation for small molecular **PTM** radicals, it is likely that not every sp^2^-carbon of the ***p*-PTM-COF** is in its radical form. Of course, quenching by π–π-stacking – often observed in these 2-dimensional COFs – is another prominent factor inhibiting emissive behavior.

Interestingly, **TTMAc-PCz-COF** has been synthesized using electropolymerisation [143]. The incorporated electron-donating unit could induce interesting optical and magnetic properties. However, neither photoluminescence nor magnetic susceptibility measurements have been performed for this material. It is to be expected that either antiferromagnetic coupling, due to conjugation between radical centers, or paramagnetic coupling for greater distances between two neighboring spin-centers will be observed.

#### Summary: triarylmethyl multiradicals

Molecular multiradicals, like diradical systems, are significantly influenced by the connectivity between the radical centers and their potential to form quinoidal or delocalized structures. In Kekulé-type multiradicals, the delocalization of unpaired spins determines the formation of open- or closed-shell structures and is affected by dihedral angles between the sp^2^-plane of the central carbons and the neighboring phenyl rings, as well as by steric effects. Increasing the distance between radical centers leads to transitions from ferro- or antiferromagnetic to paramagnetic behavior.

In covalent materials, Kekulé multiradicals often exhibit antiferromagnetic behavior, due to quinoidal structure formation, while non-Kekulé multiradicals show ferromagnetic interactions when spatial distances are small. Hydrogen-bonded radical frameworks generally display ferromagnetism or paramagnetism, with solvent molecules influencing the latter.

Predicting the magnetic properties of MOFs remains challenging, due to the influence of the metal ions on the geometry and the electronic properties. Optical properties, particularly emission and quantum yields, are understudied, with π–π stacking in 3D COFs often quenching the emissive pathways. Adjusting framework dimensions, dihedral angles, or functionalizing **TTM** with donor moieties like carbazole may enable luminescent behavior.

Magnetoluminescence, as seen in frameworks like **bisZn** and **trisZn**, stems from radical ordering and invites further investigation into whether similar mechanisms apply to other MOFs. Incorporating **TTM** radicals into non-radical matrices has been shown to enhance ϕ by mitigating self-quenching, suggesting that precisely tailored introduction of radical units into otherwise inert framework materials could reveal new insights into the interplay of magnetic and optical properties.

## Conclusion

Organic radicals with their long electron spin coherence times and diverse opportunities for functionalization, represent interesting molecular qubit materials for advanced optoelectronic applications and as novel molecular quantum materials. Their luminescence, tunable electronic ground state conformation, and their ability to be connected into 1, 2, and 3-dimensional frameworks renders trityl-based radicals interesting for quantum sensing, quantum computing, and quantum communication applications.

Whereas trityl monoradicals have been widely investigated and the effect of the donor moiety on the emission and photostability is well-understood, there remain open questions on how to implement high ϕ into diradicals and multiradicals.

While molecular color centers have been produced in metal-organic complexes [146–147], highly luminescent diradicals with a distinct triplet ground state remain unattainable to date.

While light emission is becoming an objective of active research for diradicals, it is not yet a topic for the higher dimensional assemblies of trityl radicals. If light emission of individual radical species inside of a 2D or 3D framework could be achieved, it could represent the starting point for quantum computing using organic radicals. Virtually there would be no limits in scalability for the number of qubits per framework. While electrical connectivity and readout will not be possible in a large 2D or 3D framework, optical strategies might be suitable for reading out the radical spin states.

Despite being an old molecule, after Gomberg’s first report in 1900 [31], the trityl radical represents an extremely timely class of molecules with many prospective uses in high technology applications.

## Supporting Information

File 1 Information about performed DFT calculations and photoluminescence quantum yield measurements.

## Data Availability

All data that supports the findings of this study is available in the published article and/or the supporting information of this article.
