# Supplementary material for: On the photoluminescence in triarylmethyl-centered mono-, di-, and multiradicals
Source: Beilstein J Org Chem. 2025 May 21;21:964–98. doi: 10.3762/bjoc.21.80 (PMC12117217; doi:10.3762/bjoc.21.80)
Supplement: File 1 — Information about performed DFT calculations and photoluminescence quantum yield measurements. [file Beilstein_J_Org_Chem-21-964-s001.pdf]

BEILSTEIN JOURNAL OF ORGANIC CHEMISTRY

# Supporting Information

for

# On the photoluminescence in triarylmethyl-centered mono-, di-, and multiradicals

Daniel Straub, Markus Gross, Mona E. Arnold, Julia Zolg and Alexander J. C. Kuehne

*Beilstein J. Org. Chem.* **2025,** *21,* 964–998. doi:10.3762/bjoc.21.80

## Information about performed DFT calculations and photoluminescence quantum yield measurements

License and Terms: This is a supporting information file under the terms of the Creative Commons Attribution License (https://creativecommons.org/licenses/by/4.0). Please note that the reuse, redistribution and reproduction in particular requires that the author(s) and source are credited and that individual graphics may be subject to special legal provisions.
The license is subject to the *Beilstein Journal of Organic Chemistry* terms and conditions: (https://www.beilstein-journals.org/bjoc/terms)

## DFT calculations

The theoretical calculations of PTM-PTM and *m*-PTH were performed with ORCA 5.0.3.[1] The molecular geometry optimizations were carried out with the UB3LYP functional at the def2-SVP level of theory in vacuum using dispersion correction with the Becke-Johnson damping scheme (D3BJ).[2]

The singlet-triplet energy gap ($\Delta E_{\mathrm{ST}}$) was calculated with the Yamaguchi spin decontamination correction according to the following equation:[3]

$$\Delta E_{\mathrm{ST}} = 2J = -\frac{2(E_{\mathrm{T}} - E_{\mathrm{BS}})}{\langle S^2 \rangle_{\mathrm{T}} - \langle S^2 \rangle_{\mathrm{BS}}}$$

Here, $J$ corresponds to the intramolecular exchange interaction within the diradicals, $E_{\mathrm{T}}$ and $E_{\mathrm{BS}}$ represent the total energy of the calculated triplet and broken-symmetry state and $\langle S^2 \rangle_{\mathrm{T}}$ and $\langle S^2 \rangle_{\mathrm{BS}}$ correspond to the respective expectation value of the total spin squared operator. The diradical character $y_0$ was calculated from the occupation number of the highest occupied natural orbital ($n_{\mathrm{HONO}}$) and of the lowest unoccupied natural orbital ($n_{\mathrm{LUNO}}$) according to Yamaguchi:[4]

$$y_0 = 1 - \frac{2T_0}{1 + T_0^2}$$

$$T_0 = \frac{n_{\mathrm{HONO}} - n_{\mathrm{LUNO}}}{2}$$

**Table S1:** Calculated electronic properties of PTM-PTM and *m*-PTH at the UB3LYP/def2-SVP level of theory.

| | **PTM-PTM** | ***m*-PTH** |
|---|---|---|
| $\boldsymbol{E_{\mathrm{T}}}$ [kcal mol$^{-1}$] | −8993695.883 | −7695210.997 |
| $\boldsymbol{E_{\mathrm{BS}}}$ [kcal mol$^{-1}$] | −8993695.891 | −7695210.244 |
| $\boldsymbol{\Delta E_{\mathrm{ST}}}$ [kcal mol$^{-1}$] | <0.1 | 1.6 |
| $\boldsymbol{\langle S^2 \rangle_{\mathrm{T}}}$ | 2.0418 | 2.0509 |
| $\boldsymbol{\langle S^2 \rangle_{\mathrm{BS}}}$ | 1.0423 | 1.0226 |
| $\boldsymbol{y_0}$ | 0.998 | 0.777 |

## Photoluminescence Quantum Yield

Photoluminescence quantum yields $\phi$ are determined with a Hamamatsu Quantaurus-QY (C11347) for cyclohexane solutions ($10^{-5}$ M) at room temperature (293 K).

## References


(1) F. Neese, *WIREs Comput. Mol. Sci.* **2022**, *12*, e1606. https://doi.org/10.1002/wcms.1606.

(2) a) F. Weigend, *Phys. Chem. Chem. Phys.* **2006**, *8*, 1057-1065. https://doi.org/10.1039/B508541A; b) F. Weigend and R. Ahlrichs, *Phys. Chem. Chem. Phys.* **2005**, *7*, 3297-3305. https://doi.org/10.1039/B515623H; c) S. Grimme, J. Antony, S. Ehrlich and H. Krieg, J. *Chem. Phys.* **2010**, *132*, 154104. https://doi.org/10.1002/jcc.21759; d) S. Grimme, S. Ehrlich and L. Goerigk, J. *Comput. Chem.* **2011**, *32*, 1456-1465. https://doi.org/10.1063/1.3382344.

(3) a) K. Yamaguchi, Y. Takahara and T. Fueno, *Applied Quantum Chemistry* (Dordrecht) **1986**, pp. 155- 184. https://doi.org/10.1007/978-94-009-4746-7_11. b) T.Soda et al. *Chem. Phys. Lett.* **2000**, *319*, 223-230. https://doi.org/10.1016/S0009-2614(00)00166-4.

(4) K. Yamaguchi, *Chem. Phys. Lett.* **1975**, *33*, 330-335. https://doi.org/10.1016/0009-2614(75)80169-2.
